# Supplementary material for: Barriers and opportunities to preventing residential bird–window collisions
Source: PLoS One. 2026 Feb 25;21(2):e0342330. doi: 10.1371/journal.pone.0342330 (PMC12935251; doi:10.1371/journal.pone.0342330)
Supplement: S1 Survey — (DOCX) [file pone.0342330.s001.docx]

2023 CU Bird-Window Collision Survey

Start of Block: Eligibility to participate

Q15 Community Associations for Environmental Sustainability (CAFES), Carleton University and Safe Wings Ottawa welcomes you! Thank **you for responding to our 5 minute questionnaire.**

 We want to know more about bird collisions with residential windows in Ottawa. As a first step, we are surveying Ottawa communities to find out where birds are hitting windows, if this is an issue, and what people might, or might not, be doing about it. 

 **To participate in this survey you must be a resident of the City of Ottawa and 16 years of age**.

 Are you a resident of the City of Ottawa?

- Yes (1)
- No (2)

Skip To: End of Survey If Community Associations for Environmental Sustainability (CAFES), Carleton University and Safe Win... = No

Q23 Are you at least 16 years of age?

- Yes (1)
- No (2)

Skip To: End of Survey If Are you at least 16 years of age? = No

| Page Break |  |
| --- | --- |

Q30
**Your Consent**
 
We are conducting this online survey to better understand where birds are hitting residential windows and the experience of residents in various community neighbourhoods. Students in SOCI 2180: Foundations in Community Engagement at Carleton University are working with Community Associations for Environmental Sustainability to publicize the survey and to do a summary analysis of the results for each participating Community Association. The data will be presented so that it will not be possible to identify you. This research has been cleared by Carleton University Research Ethics Board-A (Project #119817). 
 
Your participation will take the form of a 5-minute-long online questionnaire. Risks of participation are minimal but include possible emotional effects of considering the topic. Benefits of participation include providing your Community Association with local data regarding bird-window collisions. 
 
We will treat your personal information as confidential, although absolute privacy cannot be guaranteed. No information that discloses your identity will be released or published without your specific consent. Research records may be accessed by the Carleton University Research Ethics Board in order to ensure continuing ethics compliance. After the study is completed, we will retain your anonymized data for future research use.


This is a voluntary survey and you may refuse to answer any question. You are free to stop at any point. However it will not be possible to withdraw your responses once you have submitted the survey. Should you provide us with your contact information, at any point you can indicate that you are no longer interested in further correspondence.
 
If you have any questions or require more information about the study, you may contact Professor Deborah Conners at deborah.conners@carleton.ca. If you have any questions regarding the ethical conduct of this project, you may also contact the Carleton University Research Ethics Board at (613)520-2600 ext. 2517 or at ethics@carleton.ca .
 
**Do you consent to participate in this survey?**

- I consent (1)
- I do not consent (2)

Skip To: End of Survey If Your Consent   We are conducting this online survey to better understand where birds are hitting... = I do not consent

End of Block: Eligibility to participate

Start of Block: Where you live

Q17 **The next four questions will help us understand more about where birds may be colliding with windows in the City.**

 How long have you lived at your current Ottawa location?

- Less than 1 year (1)
- 1 to 2 years (2)
- 2 to 5 years (3)
- Over 5 years (4)
- I don't live in the City of Ottawa (5)

Skip To: End of Survey If The next four questions will help us understand more about where birds may be colliding with wind... = I don't live in the City of Ottawa

| Page Break |  |
| --- | --- |

| Page Break |  |
| --- | --- |

Q18 What type of housing do you live in?

- One storey house (including semi-detached and row house) (1)
- Two to four story house (including semi-detached and row house) (2)
- Apartment - 6 stories or less (3)
- Apartment - more than 6 stories (4)

| Page Break |  |
| --- | --- |

Q20 Do you have a bird feeder?

- Yes (1)
- No, but my close neighbour has a bird feeder (2)
- No (3)

| Page Break |  |
| --- | --- |

Q22 What neighbourhood do you live in?

- Participating CA 1 (1)
- CA 2 (2)
- CA 3 (3)
- CA 4 (4)
- CA 5 (5)
- Other (please specify) (6) __________________________________________________
- I don't know (7)

Skip To: End of Block If What neighbourhood do you live in? = Participating CA 1

Skip To: End of Block If What neighbourhood do you live in? = CA 2

Skip To: End of Block If What neighbourhood do you live in? = CA 3

Skip To: End of Block If What neighbourhood do you live in? = CA 4

Skip To: End of Block If What neighbourhood do you live in? = CA 5

Q9
Having your postal code will tell us your neighbourhood. 


What is your postal code?

________________________________________________________________

End of Block: Where you live

Start of Block: Demographics

| Page Break |  |
| --- | --- |

Q20
**Now, we would like to learn a little bit about you to help us know who is responding to our survey. These questions are optional.**
 
What is your age?

- 16 - 30 (1)
- 31 - 50 (2)
- 51 or older (3)
- Prefer not to say (4)

| Page Break |  |
| --- | --- |

Q4 How would you describe your gender?

- Male (1)
- Female (2)
- Transgender (3)
- You don't have an option that applies to me. I identify as: (4) __________________________________________________
- Prefer not to say (5)

| Page Break |  |
| --- | --- |

Q5 How would you describe your sexual orientation?

- Straight/heterosexual (1)
- 2SLGBTQ+ (2)
- You don't have an option that applies to me. I identify as: (3) __________________________________________________
- Prefer not to say (4)

| Page Break |  |
| --- | --- |

Q7 How would you best describe yourself?

- Indigenous (1)
- Asian (2)
- Brown (3)
- Black (4)
- White (5)
- You don't have an option that applies to me. I identify as: (6) __________________________________________________
- Prefer not to say (7)

| Page Break |  |
| --- | --- |

Q16 Do you identify as having a disability or a chronic health issue that affects your day to day life?

- Yes (1)
- No (2)
- You don't have an option that applies to me. I identify as: (3) __________________________________________________
- Prefer not to say (4)

End of Block: Demographics

Start of Block: Knowledge of Issue

Q1
**Now we would like to explore your experience with bird-window collisions.**
 
Have you ever heard about birds colliding with the windows of homes in Ottawa or not?

- Definitely yes (1)
- Probably yes (2)
- Probably not (3)
- Definitely not (4)

| Page Break |  |
| --- | --- |

Q2 Have you ever observed a bird colliding with a window in your current home or not?

- Definitely yes (1)
- Probably yes (2)
- Probably not (3)
- Definitely not (4)

| Page Break |  |
| --- | --- |

Q3 Do you think bird-window collisions at homes in Ottawa are an issue or not?

- Definitely yes (1)
- Probably yes (2)
- Probably not (3)
- Definitely not (4)

End of Block: Knowledge of Issue

Start of Block: Willingness to take action

Q9
**Now we would like to understand what you are willing or not willing to do about this situation.**
 
Are you willing or not willing to apply materials to your windows to reduce bird-window collisions?

- Definately willing (1)
- Probably willing (2)
- Probably not willing (3)
- Definately not willing (4)
- Already have (please describe actions taken) (5) __________________________________________________

End of Block: Willingness to take action

Start of Block: Barriers and possible solutions to taking action

| Page Break |  |
| --- | --- |

Q10 What stops, or would stop, you from taking action (or more action)? Check all that apply.

- Time / getting around to it (1)
- Wanting to have a clear view from windows (2)
- I don't like the look of anything on my windows (3)
- Cost (4)
- Not having the skills to apply the materials (5)
- Access to outside of windows to apply the materials (6)
- I rent and am not sure if I am allowed (7)
- Birds infrequently or never collide with my windows (8)
- Not sure what is available (9)
- I don't think this is an issue requiring action (10)
- Other (please specify) (11) __________________________________________________

| Page Break |  |
| --- | --- |

Q11 What would support you to take action?

- Someone to come and apply the materials (1)
- Application of materials I like the look of (2)
- Free materials (3)
- Clear instructions (4)
- More information (5)
- Permission from landlord (6)
- Evidence that there is a need for me to take action (7)
- Other (please specify) (8) __________________________________________________

End of Block: Barriers and possible solutions to taking action

Start of Block: Taking Action

Q12 Would you apply materials to reduce bird-window collisions if you were given a free starter kit with material to apply to a window in your home?

- Definitely yes (1)
- Probably yes (2)
- Probably not (3)
- Definitely not (4)

Skip To: End of Survey If Would you apply materials to reduce bird-window collisions if you were given a free starter kit w... = Definitely not

End of Block: Taking Action

Start of Block: Follow up

Q13 Below are some actions that you can take to get involved in reducing bird mortality from window collisions in Ottawa. Check all that you are interested in.

- I would like to receive a starter kit with materials to protect birds from one of my windows (1)
- I would like to receive information about how I can reduce bird-window collisions at my home (2)
- I would like to attend a workshop to learn more about preventing bird-window collisions (3)
- I do not want to receive any further information or materials (4)

Skip To: End of Survey If Below are some actions that you can take to get involved in reducing bird mortality from window c... = I do not want to receive any further information or materials

| Page Break |  |
| --- | --- |

Q14 That's great that you are interested. Please provide your name, email and phone number below so that we may contact you. You may also explore the Safe Wings Ottawa website at: safewings.ca

- Name (1) __________________________________________________
- Email address (2) __________________________________________________
- Phone number (3) __________________________________________________

| Page Break |  |
| --- | --- |

Q24 These are all the questions we have for you. Do you have any further comments?

________________________________________________________________

End of Block: Follow up
