# Supplementary material for: Barriers and opportunities to preventing residential bird–window collisions
Source: PLoS One. 2026 Feb 25;21(2):e0342330. doi: 10.1371/journal.pone.0342330 (PMC12935251; doi:10.1371/journal.pone.0342330)
Supplement: S1 Table — (DOCX) [file pone.0342330.s002.docx]

**S2 Table.** Results from cumulative link mixed model of relationship between willingness and age, housing type, gender, and ownership of a bird feeder. Positive parameter estimates indicate the barrier is correlated with willingness. Negative parameter estimates indicate the barrier is correlated with unwillingness. Significant relationships in bold and denoted by *.

| **Demographic Category** | **Variable** | **Estimate (± Std. Error)** | **P-value** |
| --- | --- | --- | --- |
| ***Age*** | ***Over 50*** | ***Reference (NA)*** | ***Reference (NA)*** |
|  | 16 - 30 | -0.85 ± 0.36 | **0.02*** |
|  | 31 - 50 | -0.30 ± 0.30 | 0.32 |
| ***Housing type*** | ***2-4 story house*** | ***Reference (NA)*** | ***Reference (NA)*** |
|  | Apartment 6 stories or under | 1.20 ± 0.54 | **<0.02*** |
|  | Apartment over 6 stories | -0.61 ± 0.38 | 0.11 |
|  | One story house | -0.22 ± 0.35 | 0.52 |
| ***Gender*** | ***Female*** | ***Reference (NA)*** | ***Reference (NA)*** |
|  | Male | -1.10 ± 0.29 | **<0.01***** |
| ***Ownership of birdfeeder*** | ***Owns a birdfeeder*** | ***Reference (NA)*** | ***Reference (NA)*** |
|  | Neighbor owns a birdfeeder | -0.93 ± 0.35 | **<0.01***** |
|  | Does not own a birdfeeder | -0.69 ± 0.31 | **<0.02*** |
