# Supplementary material for: Barriers and opportunities to preventing residential bird–window collisions
Source: PLoS One. 2026 Feb 25;21(2):e0342330. doi: 10.1371/journal.pone.0342330 (PMC12935251; doi:10.1371/journal.pone.0342330)
Supplement: S2 Table — (DOCX) [file pone.0342330.s003.docx]

**S3 Table.** Results from cumulative link mixed model of relationship between barriers and willingness from 312 respondents. Positive parameter estimates indicate the barrier is correlated with willingness. Negative parameter estimates indicate the barrier is correlated with unwillingness. Significant relationships (p < 0.05) are in bold and denoted by *.

| **Barrier** | **Estimate ± SE** | ***p*** |
| --- | --- | --- |
| Wanting a clear view on windows | -0.78 ± 0.29 | **<0.01**** |
| Do not perceive bird-window collisions to be an issue requiring action | -1.60 ± 0.60 | **<0.01**** |
| Not liking the look of anything on windows | -0.86 ± 0.36 | **0.02*** |
| Not observing bird-window collisions on personal windows | -0.10 ± 0.25 | 0.69 |
| Time/getting around to it | 0.22 ± 0.26 | 0.40 |
| Unclear tenancy rules | 0.04 ± 0.32 | 0.89 |
| Cost | -0.11 ± 0.29 | 0.70 |
| Access outside windows to apply materials | 0.08 ± 0.27 | 0.76 |
| Not having the skills to apply the materials | 0.17 ± 0.35 | 0.62 |
| Not knowing what is available | 0.49 ± 0.30 | 0.10 |
