# Supplementary material for: Barriers and opportunities to preventing residential bird–window collisions
Source: PLoS One. 2026 Feb 25;21(2):e0342330. doi: 10.1371/journal.pone.0342330 (PMC12935251; doi:10.1371/journal.pone.0342330)
Supplement: Attachment — Submitted filename: PONE-D-25-29481_reviewer.pdf [file pone.0342330.s004.pdf]

PLOS ONE

Barriers and opportunities to preventing residential bird-window collisions

--Manuscript Draft--

**Manuscript Number:**	PONE-D-25-29481
**Article Type:**	Research Article
**Full Title:**	Barriers and opportunities to preventing residential bird-window collisions
**Short Title:**	Preventing residential bird-window collisions
**Corresponding Author:**	Anastasia Lysyk<br>Carleton University<br>Ottawa, Ontario CANADA
**Keywords:**	bird-window collisions; pro-environmental behaviour; willingness; window treatments; participatory survey
**Abstract:**	Collisions with windows are a leading source of avian mortality in North America. Window treatment options are commercially available; however, these solutions are rarely used. To investigate knowledge and perceptions of bird-window collisions, willingness to treat windows, and barriers and solutions to treating windows we conducted a survey of residents in Ottawa, Canada. Of 422 survey respondents, 90.7% had previously heard of bird-window collisions, 58.5% had previously observed a collision, 88.0% consider collisions with windows to be an issue in Ottawa, and 87.0% were willing to treat their windows. For all survey respondents, the top barriers reducing willingness to treat windows included the perception that birds infrequently or never collide with windows (parameter estimate ± standard error, PE ± SE = -1.29 ± 0.54, p = 0.02), aesthetics (PE ± SE = -0.77 ± 0.31, p = 0.01), and wanting a clear view from windows (PE ± SE = -0.88 ± 0.25, p < 0.01). For those willing to treat their windows, lack of time was the most identified barrier (38.2%), while for those unwilling to treat their windows, the need for more evidence that bird-window collisions requires action was most identified (49.1%). Top potential solutions were provision of free materials, aesthetically pleasing materials, and clear instructions. Our results suggest that Ottawa residents are generally willing to treat their windows at home and we identify key barriers between willingness and implementation. To encourage bird-friendly window treatment at a wider scale, we suggest targeted messaging highlighting the impact of low-rise housing in driving the problem and the solution to bird-window collisions. Our results also highlight the opportunity for advocacy groups to aid residents in overcoming practical barriers to treating their windows.
**Order of Authors:**	Anastasia JV Lysyk
	Aalia I Khan
	Deborah Conners
	Rachel T Buxton
**Additional Information:**	
**Question**	**Response**
**Financial Disclosure**<br><br>Enter a financial disclosure statement that describes the sources of funding for the work included in this submission. Review the submission guidelines for detailed requirements. View published research articles from *PLOS ONE* for specific examples.	Yes

Powered by Editorial Manager® and ProduXion Manager® from Aries Systems Corporation

This statement is required for submission and **will appear in the published article** if the submission is accepted. Please make sure it is accurate.<br><br>**Funded studies**<br>Enter a statement with the following details:<br>• Initials of the authors who received each award<br>• Grant numbers awarded to each author<br>• The full name of each funder<br>• URL of each funder website<br>• Did the sponsors or funders play any role in the study design, data collection and analysis, decision to publish, or preparation of the manuscript?<br><br>Did you receive funding for this work?	
Please add funding details.<br>as follow-up to "**Financial Disclosure**<br><br>Enter a financial disclosure statement that describes the sources of funding for the work included in this submission. Review the submission guidelines for detailed requirements. View published research articles from *PLOS ONE* for specific examples.<br><br>This statement is required for submission and **will appear in the published article** if the submission is accepted. Please make sure it is accurate.<br><br>**Funded studies**<br>Enter a statement with the following details:<br>• Initials of the authors who received each award<br>• Grant numbers awarded to each author<br>• The full name of each funder<br>• URL of each funder website<br>• Did the sponsors or funders play any role in the study design, data collection and analysis, decision to publish, or preparation of the manuscript?<br><br>Did you receive funding for this work?"	Funding was provided to RTB, AJVL, and AK through three funding sources.<br><br>1) Natural Science and Engineering Research Council of Canada grant (RGPIN 04888) (https://www.nserc-crsng.gc.ca/index_eng.asp).<br>2) the Kenneth Molson Foundation (https://fondationmolson.org/en/).<br>3) Environment and Climate Change Canada (GCXE24S042) (https://www.canada.ca/en/environment-climate-change.html).<br><br>Funders did not play any role in study design, data collection and analysis, decision to publish, or preparation of the manuscript.

Please select the country of your main research funder (please select carefully as in some cases this is used in fee calculation).<br>as follow-up to "**Financial Disclosure**<br><br>Enter a financial disclosure statement that describes the sources of funding for the work included in this submission. Review the submission guidelines for detailed requirements. View published research articles from *PLOS ONE* for specific examples.<br><br>This statement is required for submission and **will appear in the published article** if the submission is accepted. Please make sure it is accurate.<br><br>**Funded studies**<br>Enter a statement with the following details:<br>• Initials of the authors who received each award<br>• Grant numbers awarded to each author<br>• The full name of each funder<br>• URL of each funder website<br>• Did the sponsors or funders play any role in the study design, data collection and analysis, decision to publish, or preparation of the manuscript?<br><br>Did you receive funding for this work?"	CANADA - CA
**Competing Interests**<br><br>Use the instructions below to enter a competing interest statement for this submission. On behalf of all authors, disclose any competing interests that could be perceived to bias this work—acknowledging all financial support and any other relevant financial or non-financial competing interests.<br><br>This statement is required for submission and **will appear in the published article** if the submission is accepted. Please make sure it is accurate and that any funding	The authors have declared that no competing interests exist.

sources listed in your Funding Information later in the submission form are also declared in your Financial Disclosure statement.<br><br>View published research articles from *PLOS ONE* for specific examples.<br><br>**NO authors have competing interests**<br><br>Enter: *The authors have declared that no competing interests exist.*<br><br>**Authors with competing interests**<br><br>Enter competing interest details beginning with this statement:<br><br>*I have read the journal's policy and the authors of this manuscript have the following competing interests: [insert competing interests here]*<br><br>* typeset	
**Ethics Statement**<br><br>Enter an ethics statement for this submission. This statement is required if the study involved:<br><br>• Human participants<br>• Human specimens or tissue<br>• Vertebrate animals or cephalopods<br>• Vertebrate embryos or tissues<br>• Field research<br><br>Write "N/A" if the submission does not require an ethics statement.<br><br>General guidance is provided below. Consult the submission guidelines for detailed instructions. **Make sure that all information entered here is included in the Methods section of the manuscript.**	This study surveyed human participants. Ethics was approved by Carleton University Research Ethics Board-B (CUREB-B #119817) on July 27, 2023. Participant consent during data collection was obtained as a response to a survey question. Data were analyzed anonymously.

**Format for specific study types**<br><br>**Human Subject Research (involving human participants and/or tissue)**<br>• Give the name of the institutional review board or ethics committee that approved the study<br>• Include the approval number and/or a statement indicating approval of this research<br>• Indicate the form of consent obtained (written/oral) or the reason that consent was not obtained (e.g. the data were analyzed anonymously)<br><br>**Animal Research (involving vertebrate animals, embryos or tissues)**<br>• Provide the name of the Institutional Animal Care and Use Committee (IACUC) or other relevant ethics board that reviewed the study protocol, and indicate whether they approved this research or granted a formal waiver of ethical approval<br>• Include an approval number if one was obtained<br>• If the study involved *non-human primates*, add *additional details* about animal welfare and steps taken to ameliorate suffering<br>• If anesthesia, euthanasia, or any kind of animal sacrifice is part of the study, include briefly which substances and/or methods were applied<br><br>**Field Research**<br><br>Include the following details if this study involves the collection of plant, animal, or other materials from a natural setting:<br>• Field permit number<br>• Name of the institution or relevant body that granted permission	
**Data Availability**<br><br>Authors are required to make all data underlying the findings described fully available, without restriction, and from the time of publication. PLOS allows rare exceptions to address legal and ethical concerns. See the PLOS Data Policy and FAQ for detailed information.	Yes - all data are fully available without restriction

A Data Availability Statement describing where the data can be found is required at submission. Your answers to this question constitute the Data Availability Statement and **will be published in the article**, if accepted.<br><br>**Important:** Stating 'data available on request from the author' is not sufficient. If your data are only available upon request, select 'No' for the first question and explain your exceptional situation in the text box.<br><br>Do the authors confirm that all data underlying the findings described in their manuscript are fully available without restriction?	
**Describe where the data may be found in full sentences. If you are copying our sample text, replace any instances of XXX with the appropriate details.**<br><br>• If the data are **held or will be held in a public repository**, include URLs, accession numbers or DOIs. If this information will only be available after acceptance, indicate this by ticking the box below. For example: *All XXX files are available from the XXX database (accession number(s) XXX, XXX.).*<br>• If the data are all contained **within the manuscript and/or Supporting Information files**, enter the following: *All relevant data are within the manuscript and its Supporting Information files.*<br>• If neither of these applies but you are able to provide **details of access elsewhere**, with or without limitations, please do so. For example:<br><br>*Data cannot be shared publicly because of [XXX]. Data are available from the XXX Institutional Data Access / Ethics Committee (contact via XXX) for researchers who meet the criteria for access to confidential data.*<br><br>*The data underlying the results presented in the study are available from (include the name of the third party*	All relevant data are within the manuscript and its Supporting Information files.

and contact information or URL).<br>• This text is appropriate if the data are owned by a third party and authors do not have permission to share the data.<br><br>* typeset	
Additional data availability information:	

Manuscript

Click here to access/download;Manuscript;barriers_opportunities_bird_windo

Barriers and opportunities to preventing residential bird-window collisions

Anastasia J.V. Lysyk[1†], Aalia I. Khan[1†], Deborah Conners[2], Rachel T. Buxton[1,3]

[1]Department of Biology, Carleton University, Ottawa, Canada

[2] Department of Sociology and Anthropology, Carleton University, Ottawa, Canada

[3]Institute of Environmental and Interdisciplinary Science, Carleton University, Ottawa, Canada

Corresponding author: Anastasia J.V. Lysyk, stashalysyk@cmail.carleton.ca

[†]Indicates shared first authorship

Author contributions:

AJVL **-** Writing – Original Draft Preparation, Writing – Review & Editing

AIK – Formal Analysis, Data Curation, Methodology, Visualization, Writing – Review and Editing

DB – Conceptualization, Data Curation, Investigation, Methodology, Project Administration, Resources, Supervision, Writing – Review and Editing

RTB – Conceptualization, Data Curation, Funding Acquisition, Investigation, Methodology, Project Administration, Resources, Supervision, Writing – Review & Editing

Abstract

Collisions with windows are a leading source of avian mortality in North America. Window treatment options are commercially available; however, these solutions are rarely used. To investigate knowledge and perceptions of bird-window collisions, willingness to treat windows, and barriers and solutions to treating windows we conducted a survey of residents in Ottawa, Canada. Of 422 survey respondents, 90.7% had previously heard of bird-window collisions, 58.5% had previously observed a collision, 88.0% consider collisions with windows to be an issue in Ottawa, and 87.0% were willing to treat their windows. For all survey respondents, the top barriers reducing willingness to treat windows included the perception that birds infrequently or never collide with windows (parameter estimate ± standard error**,** PE ± SE = -1.29 ± 0.54, p = 0.02), aesthetics (PE ± SE = -0.77 ± 0.31, p = 0.01), and wanting a clear view from windows (PE ± SE = -0.88 ± 0.25, p < 0.01). For those willing to treat their windows, lack of time was the most identified barrier (38.2%), while for those unwilling to treat their windows, the need for more evidence that bird-window collisions require action was most identified (49.1%). Top potential solutions were provision of free materials, aesthetically pleasing materials, and clear instructions. Our results suggest that Ottawa residents are generally willing to treat their windows at home and we identify key barriers between willingness and implementation. To encourage bird-friendly window treatment at a wider scale, we suggest targeted messaging highlighting the impact of low-rise housing in driving the problem and the solution to bird-window collisions. Our results also highlight the opportunity for advocacy groups to aid residents in overcoming practical barriers to treating their windows.

Introduction

Birds in North America are experiencing dramatic declines due to a variety of human-driven threats (1). Each year in North America, billions of birds are killed by colliding with windows, making it one of the largest sources of direct avian mortality (2,3). Several studies and grassroots programs have quantified the magnitude of the issue of bird-window collisions. Citizen science programs in multiple countries led by members of the public who patrol cities for birds that collide with windows have demonstrated the dramatic numbers of annual bird-window collisions (3). This includes the Fatal Light Awareness Program (FLAP), that has documented over 100,000 bird-window collisions across the globe (4). The resulting data have been used in research, successful lawsuits, advocacy programs, and changes to municipal green standards in Canada (5) and beyond (3,6).

Birds collide with windows because they do not perceive them as barriers in the environment due to the transparent and reflective properties of glass (7). Solutions like window treatments are now commercially available to prevent bird-window collisions, including window films and decals (8). Patterns of uniformly spaced visual markers that cover a window surface are known to provide a barrier that results in avoidance by birds (8). Efficacy of these visual patterns has been found to range from 92-95% reduction in collisions at low-rise buildings (9,10) and by 84% at a rural residential building (11).

While bird-window collisions occur at all types of buildings, houses account for approximately 90% of all collisions, as they are relatively more abundant than tall commercial buildings (2). Some conservative estimates indicate window-related mortality rates at residences are between 1.2 and 2.4 birds annually at Canadian single-and semi-detached houses (12). Other

studies have estimated collision mortalities to be 0.3-15.7 birds per year per residence (2) and reported upwards of 16-43 annual collisions for the worst residences (13). Although many people have observed bird-window collisions at their homes (39% of respondents in (13)), awareness and uptake of preventative measures remains rare (14).

Despite an understanding of the magnitude of the problem and the effectiveness of solutions, there have been few coordinated efforts to address the bird-window collisions at scale in Canada and elsewhere (14). Treating windows can be a contentious issue, as glass is valued for its aesthetically appealing transparency that allows landscape views (15). Retro-fitting windows can be cost-prohibitive and logistically challenging, requiring adhering decals to the outside surface of sometimes difficult-to-reach windows. Although bird mortality resulting from window collisions are unlawful in Canada under the *Migratory Birds Convention Act, 1994* (16), enforcement typically focuses urban commercial buildings despite residential homes being a key source of bird-window collisions (2,13). Given the potential for window treatments at residential homes to reduce up to 90% of bird-window collisions, it is important to understand how to motivate residents to implement bird-window protection measures.

In a survey of the Canadian public, one study found that Canadian's willingness to pay to reduce bird-window collisions at their home was positively influenced by respondents' interest in birds, previous donations to conservation organizations, and demographic factors like age and income (12). In another study exploring perceptions of bird-window collisions in the US, architects, homeowners, and conservation practitioners indicated a general receptivity to management measures (17). However, despite awareness and receptivity, most people do not understand the magnitude of the issue nor available solutions or resources (15).

Community science programs to raise awareness about bird-window collisions have proliferated across North America (3), including local awareness-raising campaigns about window treatments (18). Strategies include increasing knowledge and public information, assuming that the more informed people are, the more likely they are to take action (19). However, many factors influence the likelihood of people engaging with conservation behaviours, including relationships to nature, social norms, and perceived ability for one's actions to be impactful (19,20). To guide efforts to increase uptake of bird-friendly window treatments, we aimed to investigate key barriers identified by residents in Ottawa to treat their windows. Specifically, our objectives were to: 1) explore the current knowledge, observation, and perception of bird-window collisions in Ottawa residents, 2) explore willingness to take action to reduce bird-window collisions and how this is influenced by knowledge, observation, and perception of bird-window collisions, and 3) explore barriers to taking action to reduce window collisions and potential solutions to these barriers. We discuss ways that knowledge of these barriers can inform broader campaigns for residents to treat windows.

Methods

Data was collected for this project in the fall of 2023 through an undergraduate social science course – SOCI/ANTH2180A: Foundations in Community Engagement in the Department of Sociology and Anthropology at Carleton University in Ottawa, Canada. Ethics were approved by Carleton University Research Ethics Board-B (CUREB-B #119817) on July 27, 2023.

Study area

Ottawa is an important stopover site for migrating birds, bordering the Great Lakes-St. Lawrence lowlands. It is among the most heavily urbanized regions of Ontario, representing critical habitat for many species of conservation concern in Canada (21). There is an active local community science program, Safe Wings Ottawa, that collects bird window-collision data and undertakes community outreach. Safe Wings estimates that approximately 250,000 birds die from colliding with windows each year in Ottawa (18). An Ottawa network, Community Associations for Environmental Sustainability (CAFES), has engaged with this issue in the past. Through CAFES, 4 community associations were involved with this study: Fisher Heights and Area Community Association, the Glebe Community Association, the Hintonburg Community Association, and the Westboro Community Association.

Data Collection

To address our research questions, we designed a survey in collaboration with Safe Wings Ottawa and CAFES. The survey was created using Qualtrics (Qualtrics, Provo, UT)(22). Undergraduate students distributed the survey by sharing QR codes, advertisements in the community, and direct engagement with the public at community centres, coffee shops, and other venues around Ottawa. Most direct engagement to circulate the survey was done in four neighborhoods in Ottawa (Fisher Heights, Hintonberg, the Glebe, and Westboro) through the relevant community associations. Responses were collected between September 21 - October 30, 2023. We limited survey responses to community members that were at least 16 years of age and lived in Ottawa. Respondents were provided a question to confirm their informed consent to participate in the survey. Upon completion of the survey, respondents were invited to a workshop hosted by CAFES to further learn about bird-window collisions and receive a free starter kit of materials to treat their windows at home.

The survey consisted of 22 questions to collect information on home traits, demographics (optional), experience with bird window-collisions (including whether respondents had knowledge of the issue, observed a bird-window collision, or perceive bird-window collisions to be an issue), willingness to act to prevent bird-window collisions, and barriers and solutions to acting. Most questions were closed on a 5-point Likert scale from “Definitely yes” to “Definitely no”. Knowledge of the issue of bird-window collisions was determined with the question: “Have you ever heard about birds colliding with the windows of homes in Ottawa or not?”. Observation of bird-window collisions was determined with the question: “Have you ever observed a bird colliding with a window in your current home or not?”. Perception of bird-window collisions as an issue was determined with the question: “Do you think bird-window collisions at homes in Ottawa are an issue or not?”. These three factors (knowledge, observation, and perception) were considered together as “awareness” to investigate the scope of people’s understanding of bird-window collisions.

Willingness to prevent bird-window collisions by treating windows was determined with the question: “Are you willing or not willing to apply materials to the outside of your windows to reduce bird-window collisions?” and used a Likert scale from “Definitely willing” to “Definitely not willing”. To assess barriers to taking action to prevent bird-window collisions we asked, "What stops, or would stop you from taking action (or more action)?" and allowed respondents to choose as many barriers from a list of 10 (e.g., time/getting around to it; cost) and/or "other" with the opportunity to specify in a text box. Finally, to explore solutions to barriers preventing action we asked, “What would support you to take action?” and allowed respondents to select from a list of 7 (e.g., someone to come and apply the materials; free materials) and/or “other” with the opportunity to specify in a text box. The full survey can be found in the supplementary

material (S1 Survey). The list of barriers and solutions to preventing bird-window collisions were generated by experts at Safe Wings Ottawa and through a review of the literature.

Data Analysis

All analyses were performed in R statistical software (v.2024.12.0; R Core Team, 2024)(23), and figures were made using the *ggplot2* package (24). We assessed the correlation between knowledge (i.e., hearing about) of bird-window collisions, observation (i.e., seeing) of bird-window collisions, perception of bird-window collisions as an issue, and willingness to act on bird-window collisions using Spearman's correlation coefficient using the *Hmisc* package (25) given the ordinal nature of the data. The resulting correlation matrix figure was created using *ggcorrplot* (26).

To explore the relationship between willingness and the frequency of each barrier, we ran a cumulative link mixed effect model with Laplace approximation, using the *ordinal* package (27). We included willingness as the response variable with the absence or presence of a barrier, age, gender, housing type, and ownership of a birdfeeder as covariates, and years spent in Ottawa as a random effect. A Spearman's correlation was conducted to test for multicollinearity prior to building the model; no covariates had correlation coefficients over 0.5.

To determine which barriers correlated most closely with different solutions selected by respondents, we calculated the Cramer's V correlation coefficient using the *vcd* package (28). Cramer's V was determined to be the best correlation test for this scenario, as it works for nominal or binary variables that form more complex (i.e., greater than 2x2) contingency tables (29). All de-identified data and code will be made available upon publication at an OSF repository.

Results

We received a total of 465 survey responses, of which 422 were complete and included in further analysis. The majority (90.4%) of respondents had lived in Ottawa for at least one year with 61.6% having lived in Ottawa for over 5 years. The largest proportion of respondents were women (71.6%), older adults (51+) (37.0%), and people who identify as ethnically white (75.7%). The second largest group of respondents were women aged 31-50 (n = 103; 22.2% of all respondents), of which, the majority were also white (n = 85). Most residents (61.9%) lived in a two to four storey house, followed by a one storey house (17.0%), an apartment with greater than 6 stories (12.4%), or an apartment with fewer than 6 stories (8.6%). About half of respondents (55.8%) had a bird feeder. Most survey respondents (66.5%) lived in the four core neighborhoods where surveys were distributed, the other 33.5% lived in a range of neighborhoods across Ottawa.

Awareness of bird-window collisions

Most survey respondents were aware of bird-window collisions, had previous knowledge of bird-window collisions and perceived it to be an issue to some extent. 90.7% of respondents had previous knowledge of bird-window collisions and 88.0% perceive window collisions an issue in Ottawa despite only 58.5% of respondents having observed a window collision before (Fig 1).

Fig 1: Proportions of 422 survey responses "definitely not", "probably not", "probably yes", and "definitely yes" to questions corresponding to observation, knowledge, perception and

willingness to address bird-window collisions in Ottawa, Canada. Those who responded "already have" for willingness to address bird-window collisions were grouped with "definitely yes".

We investigated correlations between knowledge, observation, and perception and the willingness of respondents to address bird window collisions at their residences (Fig 2). All correlations were statistically significant ($p < 0.05$). Previous knowledge of window collisions was correlated with perception of window collisions as an issue ($r = 0.38$, $p < 0.01$). However, more than half of respondents that had not heard of window collisions still perceived them to be an issue (60.5%). Knowledge of window collisions was also correlated with observation of a bird-window collision ($r = 0.33$).

Fig 2. Spearman's rank-order correlation test results for respondents' knowledge (i.e., whether respondents had heard of bird-window collision issues), observation (i.e., whether respondents have observed a bird-window collision), perception (i.e., whether they perceive bird-window collision to be an issue) and willingness (i.e., willingness to act on bird-window collision issues) in Ottawa, Canada.

Willingness to address bird-window collisions

Most survey respondents were willing to treat their windows (76.0%) and only 9.2% of respondents identified they had already treated their windows (Fig 1). Of the willing respondents, 89.7% of them also consider bird-window collisions to be an issue and 40.2% had observed a bird-window collision before. Of respondents who were not willing to treat their windows, 17.5% had not heard of bird-window collisions, 54.4% had not observed a bird-window collision, and 36.8% do not think bird-window collisions are an issue in Ottawa. 9.2% of respondents had already treated their windows.

Perception of bird-window collisions as an issue had the strongest relationship to willingness (Fig 2) ($r = 0.41$; $p < 0.01$). Knowledge of window collisions was moderately related to willingness ($r = 0.23$; $p < 0.01$). Of survey respondents who were knowledgeable about window collisions, 88.1% were willing or had already treated their windows. Of those that were not knowledgeable, 76.3% were still willing to treat their windows. Previous observation of a collision had the weakest relationship to willingness ($r = 0.16$; $p < 0.01$). However, of those that had not seen a window collision, 81.3% were still willing or had already taken action to treat their windows. Of those who had seen a window collision, 87.8% were willing or had already taken action to treat their windows.

Demographic variables also affected willingness. Our cumulative link mixed model (Fig 3, numerical results in S2 Table) found that respondents who selected Male as their gender were significantly less willing to treat windows than those who selected Female (PE ± SE = -1.00 ± 0.24, $p < 0.01$). Compared to respondents aged 50+, those aged 31-50 were significantly less willing to treat their windows (PE ± SE = -0.56 ± 0.25, $p = 0.03$), and those aged 16-30 were very significantly less willing to treat their windows (PE ± SE = -1.23 ± 0.30, $p < 0.01$). Owning a birdfeeder also affected willingness, where respondents without a birdfeeder were significantly less willing to treat their windows (PE ± SE = -0.67 ± 0.26, $p < 0.01$) than respondents who owned a birdfeeder. Participants whose neighbours owned a birdfeeder, while they themselves did not, were even less willing to treat their windows (PE ± SE = -1.07 ± 0.29, $p < 0.01$). When it came to housing type, respondents who resided in a one storey house demonstrated a similar amount of willingness to those who resided in a 2-4 storey house. Those who resided in apartments over 6 storeys were slightly less willing to treat their windows, although not significantly (PE ± SE = -0.61 ± 0.35, $p = 0.08$). However, those who resided in apartments

between 4 and 6 storeys were significantly more willing to treat their windows (PE ± SE = 1.20 ± 0.40, $p < 0.01$).

Fig 3. Forest plot of results from cumulative link mixed model of relationship between willingness and age, housing type, gender, and ownership of a bird feeder from 422 survey respondents from Ottawa, Canada. Points depict effect estimates with error bars depicting 95% confidence interval. "Gender: Transgender" and "Gender: Non-binary" had too few responses for confidence intervals.

Barriers to action

For all respondents, the most identified barrier to treating windows was that birds infrequently or never collide with their windows (33.9%), followed by time (33.5%). Other commonly identified barriers included wanting to have a clear view from windows (27.3%), cost (25.4%) and access to the outside of windows to apply materials (24.9%) (Fig 4).

Fig 4: Number of responses to each barrier selected by 422 respondents from Ottawa, Canada. Responses are separated by those who are not willing, willing, and already have acted to address bird-window collisions. Multiple barriers could be selected by each respondent.

For respondents that are willing to treat their windows, time was the largest barrier (38.2%) and the perception that birds infrequently collide with their windows was the second most identified (36.0%). For respondents willing to treat their windows, only 3.4% identified they did not think bird-window collisions were an issue requiring action. For those not willing to treat their windows, birds infrequently or never colliding with their windows was the most identified barrier (45.6%), followed by wanting to have a clear view from their windows

(42.1%). Cost, unclear tenancy rules, and not liking the look of anything on their windows were other commonly identified barriers by those not willing to treat their windows.

While birds infrequently colliding with respondents' windows and lack of time were the most commonly selected barriers, they did not have a significant influence on willingness. Barriers with significant negative effects on willingness were aesthetics (those not liking the look of anything on their windows) (PE ± SE = -0.77 ± 0.31, $p = 0.01$), not perceiving window collisions an issue requiring action (PE ± SE = -1.29 ± 0.54, $p = 0.02$), and wanting a clear view from their windows (PE ± SE = -0.88 ± 0.25, $p < 0.01$). Model results suggest these barriers are negatively related to willingness, where respondents that value aesthetics, wanting a clear view from their windows, and not perceiving this an issue needing action are significantly less willing to take action to treat their windows (Fig 5, numerical results in S3 Table).

Fig 5. Forest plot of results from cumulative link mixed model of relationship between willingness and barriers selected by respondents from Ottawa, Canada. Points depict effect estimates with error bars depicting 95% confidence interval.

Solutions to barriers

Amongst all respondents, providing free materials is the most commonly selected way to support people treating their windows (42.8%) (Fig 6). Aesthetically pleasing materials (31.6%, selected as "Application of materials I like the look of"), being given clear instructions (27.3%), and having someone else apply materials for them (26.7%) were also identified as strong sources of support. For those willing to treat their windows, more information was also important (29.7%). For those not willing to treat their windows, evidence of the need to take action was the

most frequently identified solution to support action (49.1%), followed by free materials (31.6%) and aesthetically pleasing materials (31.6%).

Fig 6. Types of solutions selected by 422 respondents from Ottawa, Canada. Responses are separated by those who are not willing, willing, and already have acted to address bird-window collisions.

We found that many of the strongest correlations between barriers to action and solution to take action for bird-window collisions identified by respondents were those where the presented solution directly addressed a specific barrier (e.g., unclear tenancy rules and receiving landlord permission, Cramer's V = 0.8 ; wanting a clear view on windows and aesthetically pleasing materials, Cramer's V = 0.42) (Fig 7). Other strong correlations (Cramer's V > 0.25) (29) included lack of awareness of available resources and clear instructions (Cramer's V = 0.35), and lack of time and provision of free materials (Cramer's V = 0.39).

Fig 7: Cramer's V correlation coefficient showing which solutions are most often associated with barriers identified by 422 respondents from Ottawa, Canada. Respondents were able to select multiple barriers and solutions each.

Discussion

Given high bird mortality caused by window collisions at residential homes, encouraging residents to treat their windows is an important bird conservation intervention. As such, understanding what factors influence people's behaviours in treating their windows can help overcome barriers and mobilize action. Our survey respondents represent Ottawa residents that are both knowledgeable about bird-window collisions and willing to treat their windows. Despite overall awareness and willingness, very few respondents had already treated their windows. Key

barriers respondents identified as preventing them from treating windows were the perception that birds infrequently collide with their windows and a lack of time, but free and aesthetically pleasing materials with clear application instructions were potential solutions. Additionally, despite many respondents having previously heard of window collisions and perceiving it to be an issue, many identified they need more evidence that this is an issue requiring action.

Awareness and willingness to take action to prevent bird-window collisions

Respondents to our survey were predominantly older white females and identified a high awareness and willingness to take action to address bird-window collisions in Ottawa. Pro-environmental behaviours are influenced by personality, attitudes, knowledge of the issue and available actions, and perceptions or knowledge of behaviour efficacy (20,30). Motivation and willingness are also influenced by socioeconomic and demographic context (31). Our results found that respondents identifying as female and those over the age of 50, were significantly more likely to be willing to treat their windows than respondents identifying as male and those between the ages of 16-50. This is supported by other studies which have found that those more likely to engage with pro-environmental behaviours are typically older, white females (32). Additionally, respondents without birdfeeders were significantly less willing to treat their windows compared to those with feeders, supporting a study by Cooper et al. (33) which found greater pro-environmental behaviour among those who engage with bird-related nature recreation activities. Respondents were generally aware of bird-window collisions and most had some knowledge of the issue and thought bird-window collisions are an issue. Despite representing a group of people that are considered more likely to take action (20), only 9.2% of

respondents had already treated their windows, suggesting barriers still exist preventing people from taking action. Our survey responses indicated all awareness factors of bird-window collisions were correlated with willingness to address bird-window collisions. Of the three awareness factors, perception that window collisions are an issue was the factor most strongly correlated with willingness (Fig 2).

Typically, efforts to share knowledge of an issue are done under the assumption that being knowledgeable makes people more likely to act (19). However, our results suggest that knowledge, while important, may not be the most influential factor in residents treating their windows. Knowledge was strongly correlated with willingness, but less so than perception that bird-window collisions are an issue. Additionally, knowledge was correlated with observation of a window collision, but it is difficult to discern if people who already know about collisions are then more likely to have observed one or if observation of a collision elicits learning of the issue. While knowledge and perception were correlated with each other, we found that some people perceived window collisions as an issue without having previous knowledge, suggesting that perception is more influential in willingness to take action. These results are similar to Knapp et al. (32) who investigated pro-environmental behaviours around homeowners and pollinators and found that knowledge was less important than perception in influencing people taking action at their homes. As survey respondents were generally willing to treat their windows, supporting those who are knowledgeable of bird-window collisions with more information to solidify their perception of bird-window collisions as an issue may improve their willingness to take action at their homes.

Current messaging and community science programs aiming to prevent bird-window collisions tend to focus on the issue within a downtown core, highlighting high-rise commercial

buildings as primary sources of window collisions and targets for mitigation efforts (2,34). In Ontario, some municipal bird-safe guidelines have begun to address this problem when constructing new commercial or industrial buildings, but it is difficult to regulate bird-safe design at homes (35). High rise buildings account for more collisions per building than low-rise buildings and present high-profile cases like McCormick Place in Chicago. In fall 2023 under 1,000 birds died overnight from colliding with McCormick Place and this event garnered public support that was successful in treating windows of this building (6). However, annual Canadian estimates of collision mortalities attribute only around 1% of cumulative collisions to high-rise buildings in comparison to the 90% of collisions from residential buildings (2). While these high-profile cases are of great importance in raising awareness to prevent collisions, they may lead to an under-emphasis on the role of low-rise buildings in contributing to bird-window collisions. This may lead to people believing bird-window collisions are not particularly an issue at homes and addressing the issue is more the responsibility of others, which has been found in other collision perception studies (17). Further research could assess people's breadth of knowledge on the issue of window collisions at home versus other buildings to explore if this is driving the gap between willingness and taking action to prevent bird-window collisions for those who are knowledgeable.

While perceiving collisions to be an issue was positively correlated with both previous knowledge and willingness to act, respondents did not need to have previous knowledge to perceive bird-window collisions as an issue. It is possible that people do not need to be knowledgeable about specific bird conservation issues to have a sense of caring or responsibility to protect birds for their inherent value (17). The desire of an individual to engage with pro-environmental behaviours can additionally be influenced by their perception of personal

responsibility or sense of obligation to act (20). In the case of bird-window collisions, the experience of observing a collision at their homes could influence people's sense of obligation as the consequences of inaction are tangible and can elicit a strong emotional reaction. However, survey responses showed that previously observing a window collision at home had the weakest correlation with willingness to address bird-window collisions suggesting that for survey respondents, a sense of personal responsibility or obligation may not arise from observing a collision.

Barriers to action and potential solutions

The most common barrier to taking action to prevent bird-window collisions amongst all respondents was the perception that birds infrequently or never collide with their windows. However, there was no significant relationship between perception that birds infrequently or never collide with windows and respondents' willingness to treat windows. This may indicate that for those who are willing to treat their windows, the issue may not be considered pressing enough in relation to their own homes to warrant the cost, time, or effort to take action. This may again relate to the fact that even if residents are knowledgeable about the issue of bird-window collisions, they may not understand the magnitude of the issue, particularly at residential homes (15,17). While many residents believe that birds infrequently or never collide with their windows, this may not reflect the true rates of collisions at homes, as most residential collisions are not witnessed (36). Collisions are a sudden event followed by predation of the carcass, and can be easily missed, especially given that many people are not in their homes for most of the day. Additionally, many homeowners have no reason to be looking for evidence of collisions, and if they are, many collisions do not result in visible evidence to find like a carcass or feathers on a window (13,36,37). Messaging strategies aiming to communicate the problem of collisions

at homes and the potential for residents to contribute to solutions could potentially help overcome this barrier.

Personal values were also a common barrier identified by respondents, where many wanted a clear view from windows and identified aesthetically pleasing materials as a solution. Previous studies have also found that homeowners value unobstructed views and are less willing to retrofit their windows if glass aesthetics are affected (12,15,38). This may be influenced by social norms and practices, or lack there-of, around bird-friendly window treatments. As they are not common on residential buildings, there may be little social pressure to implement window treatments. However, while people value clear views from their windows, Sheng et al. (38) noted that survey respondents would be willing to compromise the view if they knew the designs would prevent bird fatalities.

Practical barriers were also identified by respondents as preventing them from treating their windows. Time was the most common, but cost, access, and tenancy rules were also identified as barriers by those willing to treat their windows. Practical solutions addressing these issues included communicating clear instructions for window treatment options that are low cost and low time investment or providing free materials and/or services to treat windows for respondents to tackle time, access, or ability barriers (Fig 4.). In general, our survey group represents people that would be more willing to engage with pro-environmental behaviours. Other studies have found that a similar demographic is also more willing to pay for environmental solutions (12,39) but cost still presented a barrier for respondents in treating their windows. Along with provision of free materials as a top solution to overcome this barrier, most survey respondents communicated they would be willing to apply window treatments if a free kit

was given to them. Financial aid is a broader solution to help address this problem, for example, monetary incentives (12) or government subsidies (17).

Limitations and future directions

Our survey focused on four specific neighbourhoods in Ottawa, with some broader participation around the city. Many responses came from residents that participate with a community association and may be more likely to be interested in taking action regarding environmental issues that other residents. Thus, applying our findings to the entire city or more broadly to other cities in Canada should be done with caution. However, our survey provides one of the first to our knowledge to explore the relationships between awareness, willingness, and barriers and solutions for home residents in Canada to make their windows bird-friendly and, with over 400 responses, offers valuable insights.

We took a broad approach to explore barriers to taking action to prevent bird-window collisions. As such, we did not assess more detailed influences of different factors on responses. For instance, we did not assess the type of knowledge people had of window collisions at their homes, nor did we assess their knowledge of window collision treatments. Additionally, the only preventative action we explored was window treatments rather than including actions such as relocating bird feeders or altering landscaping at homes (12,14), advocating for bird-friendly guidelines in cities, or contributing donations to organizations that work with window collisions. These would make interesting avenues for further research to motivate action to combat bird-window collisions.

Potential pathways and opportunities for increasing action

While our results indicate the importance of knowledge in influencing willingness to take action on bird-window collisions, they also suggest a shift in the type of knowledge presented by conservation organizations to emphasize the importance of residential homes in the problem and solution to preventing collisions. Survey respondents identified the need for more evidence that bird-window collisions are an issue requiring action at their own homes. Survey respondents were willing to address bird-window collisions and perceive it as a problem in Ottawa. However, most wanted more information about the issue at their homes, suggesting people care about bird-window collisions but think that their actions at home would not necessarily be impactful. This is useful to collision advocacy groups to tailor messaging to highlight the prevalence of bird-window collisions at low-rise buildings and homes. Targeted messaging should emphasize that individual actions of treating windows at homes with effective treatments can avoid a few collisions, yet when scaled up will have a enormous impact on reducing bird-window collisions. This messaging could contribute to overcoming barriers related to aesthetics and values related to the view from windows if people felt the outcomes of their actions outweighed these barriers (as indicated by Sheng et al. (38)). Additionally, providing information of different types of window treatments and using specific supporting evidence that these reduce collisions has the potential to improve people's behaviours in treating their windows.

Given the results of our survey identifying access to windows, cost and time as barriers, another potential opportunity for advocacy groups is to make treating home windows as easy as possible for residents (e.g., identifying window washing companies that treat windows and providing volunteer services to treat windows, e.g., (40)). Addressing bird-window collisions requires effort compared to other biodiversity conservation actions that involve inaction (e.g., reduced lawn mowing to support urban pollinators). Our results suggest efforts to make treating

windows an easier process to accomplish, aesthetically pleasing, and connected to peoples' values and sense of responsibility, would help bridge the gap between willingness and taking action to prevent bird-window collisions.

Acknowledgements

We would like to thank A. Keller-Herzog, P. MacDonald, and others at the Community Associations for Environmental Sustainability, J. Niwa and W. English at Safe Wings Ottawa, and D. Doherty at Bird-friendly Ottawa for supporting this project. We thank community association representatives W. Burpee and P. MacDonald (FHACA), J. Freeman and T. Beauchamp (CGA), R. Philar (HCA), and D. Chapman (WCA), and the SOCI/ANTH 2180 class for recruiting survey respondents. RTB, AJVL, and AK were funded by NSERC (RGPIN 04888), the Kenneth Molson Foundation, and Environment and Climate Change Canada (GCXE24S042).

References

1. Rosenberg KV, Dokter AM, Blancher PJ, Sauer JR, Smith AC, Smith PA, et al. Decline of the North American avifauna. Science. 2019 Oct 4;366(6461):120–4.

2. Machtans C, Wedeles C, Bayne E. A First Estimate for Canada of the Number of Birds Killed by Colliding with Building Windows. Avian Conservation and Ecology [Internet]. 2013 Sep 30 [cited 2023 Dec 5];8(2). Available from: https://www.ace-eco.org/vol8/iss2/art6/

3. Loss SR, Will T, Loss SS, Marra PP. Bird–building collisions in the United States: Estimates of annual mortality and species vulnerability. The Condor. 2014;116(1):8–23.

4. FLAP Canada. Global bird collision mapper [Internet]. 2022. Available from: https://birdmapper.org/app/

5. City of Toronto. Bird-friendly guidelines [Internet]. 2021. Available from: https://www.toronto.ca/city-government/planning-development/official-plan-guidelines/design-guidelines/bird-friendly-guidelines/

6. Farnsworth A, Horton KG, Marra PP. To mitigate bird collisions, enforce the Migratory Bird Treaty Act. Proceedings of the National Academy of Sciences. 2024;121(9):2320411121.

7. Klem D. Bird: window collisions. The Wilson Bulletin. 1989;606–20.

8. Klem D, Saenger PG. Evaluating the effectiveness of select visual signals to prevent bird-window collisions. The Wilson Journal of Ornithology. 2013;125(2):406–11.

9. Brown BB, Santos S, Ocampo-Peñuela N. Bird-window collisions: Mitigation efficacy and risk factors across two years. PeerJ. 2021;9:e11867.

10. De Groot KL, Wilson AG, McKibbin R, Hudson SA, Dohms KM, Norris AR, et al. Bird protection treatments reduce bird-window collision risk at low-rise buildings within a Pacific coastal protected area. PeerJ. 2022;10:13142.

11. Ocampo-Peñuela N, Peñuela-Recio L, Ocampo-Durán Á. Decals prevent bird-window collisions at residences: a successful case study from Colombia. Ornitología Colombiana. 2016;15.

12. Warren EJ. A clear threat to conservation: using public policy to reduce bird collisions with windows in homes. 2013.

13. Bayne EM, Scobie CA, Rawson-Clark M. Factors influencing the annual risk of bird–window collisions at residential structures in Alberta, Canada. Wildlife Research. 2012;39(7):583–92.

14. Klem D. Landscape, legal, and biodiversity threats that windows pose to birds: A review of an important conservation issue. Land. 2014;3:351–61.

15. Menacho-Odio RM. Local perceptions, attitudes, beliefs, and practices toward bird-window collisions in Monteverde, Costa Rica. UNED Research Journal [Internet]. 2018 Feb 28 [cited 2024 Jul 10];10. Available from: https://revistas.uned.ac.cr/index.php/cuadernos/article/view/2038

16. Migratory Birds Convention Act. S.C. 1994, c. 22 1994.

17. Riggs GJ, Joshi O, Loss SR. Stakeholder perceptions of bird-window collisions. Plos one. 2022;17(2):0263447.

18. Safe Wings Ottawa. Safe Wings Ottawa [Internet]. 2025. Available from: https://safewings.ca/

19. Kidd LR, Garrard GE, Bekessy SA, Mills M, Camilleri AR, Fidler F, et al. Messaging matters: A systematic review of the conservation messaging literature. Biological conservation. 2019;236:92–9.

20. Hines JM, Hungerford HR, Tomera AN. Analysis and synthesis of research on responsible environmental behavior: A meta-analysis. The Journal of environmental education. 1987;18(2):1–8.

21. Pogson A, Dennison CJ, Khan AI, Raposo M, Mohns ML, Davy CM, et al. The importance of cities in protecting imperiled species.

22. Qualtrics [Internet]. Provo, UT: Qualtrics; 2025. Available from: https://www.qualtrics.com

23. R Core Team. R: A language and environment for statistical computing [Internet]. Vienna, Austria: R Foundation for Statistical Computing; 2024. Available from: https://www.R-project.org

24. Wickham H. ggplot2: Elegant Graphics for Data Analysis. Springer-Verlag New York; 2016.

25. Harrell Jr F. Hmisc: Harrell Miscellaneous [Internet]. 2025. Available from: https://CRAN.R-project.org/package=Hmisc.

26. Kassambara A. ggcorrplot: Visualization of a Correlation Matrix using “ggplot2” [Internet]. 2023. Available from: https://CRAN.R-project.org/package=ggcorrplot.

27. Christensen R. ordinal-Regression Models for Ordinal Data [Internet]. 2023. Available from: https://CRAN.R-project.org/package=ordinal.

28. Meyer D, A. Z, Hornik K. The Strucplot Framework: Visualizing Multi-Way Contingency Tables with vcd. Journal of Statistical Software. 2006;17(3):1–48.

29. Akoglu H. User's guide to correlation coefficients. Turkish journal of emergency medicine. 2018;18(3):91–3.

30. Frick J, Kaiser FG, Wilson M. Environmental knowledge and conservation behavior: Exploring prevalence and structure in a representative sample. Personality and Individual differences. 2004;37(8):1597–613.

31. Asah ST, Blahna DJ. Practical implications of understanding the influence of motivations on commitment to voluntary urban conservation stewardship. Conservation Biology. 2013;27(4):866–75.

32. Knapp JL, Phillips BB, Clements J, Shaw RF, Osborne JL. Socio-psychological factors, beyond knowledge, predict people's engagement in pollinator conservation. People and Nature. 2021;3(1):204–20.

33. Cooper C, Larson L, Dayer A, Stedman R, Decker D. Are wildlife recreationists conservationists? Linking hunting, birdwatching, and pro-environmental behavior. The Journal of Wildlife Management. 2015;79(3):446–57.

34. Loss SR, Li BV, Horn LC, Mesure MR, Zhu L, Brys TG, et al. Citizen science to address the global issue of bird–window collisions. Frontiers in Ecology and the Environment. 2023;21(9):418–27.

35. Samuels B. Ontario government considers a Bird-Safe Windows Bill. Ontario Field Ornithologists News. 2024;42(1):12–3.

36. Samuels B, Fenton B, Fernández-Juricic E, MacDougall-Shackleton SA. Opening the black box of bird-window collisions: passive video recordings in a residential backyard. PeerJ. 2022 Dec 20;10:e14604.

37. Kornreich A, Partridge D, Youngblood M, Parkins K. Rehabilitation outcomes of bird-building collision victims in the Northeastern United States. Plos one. 2024;19(8):0306362.

38. Sheng GQ, Ingabo SN, Chan YC. Evaluating the impact of bird collision prevention glazing patterns on window views. Building and Environment. 2024;259:111657.

39. Eylering A, Büscher M, Funk M, Boldt J, Fiebelkorn F. Willingness of the German population to donate toward bird conservation: An application of the protection motivation theory. Global Ecology and Conservation. 2022;38:02176.

40. Safe Wings Ottawa. Suppliers and Installers [Internet]. 2025. Available from: https://safewings.ca/solutions/suppliers-and-installers/

Supporting Information

S1 Survey. Qualtrics survey questionnaire on bird-window collisions for residents of Ottawa, Canada.

S1 Table. Results from cumulative link mixed model of relationship between willingness and age, housing type, gender, and ownership of a bird feeder.

S2 Table. Results from cumulative link mixed model of relationship between barriers and willingness.

Figure 1

Click here to access/download;Figure;Fig1.tif

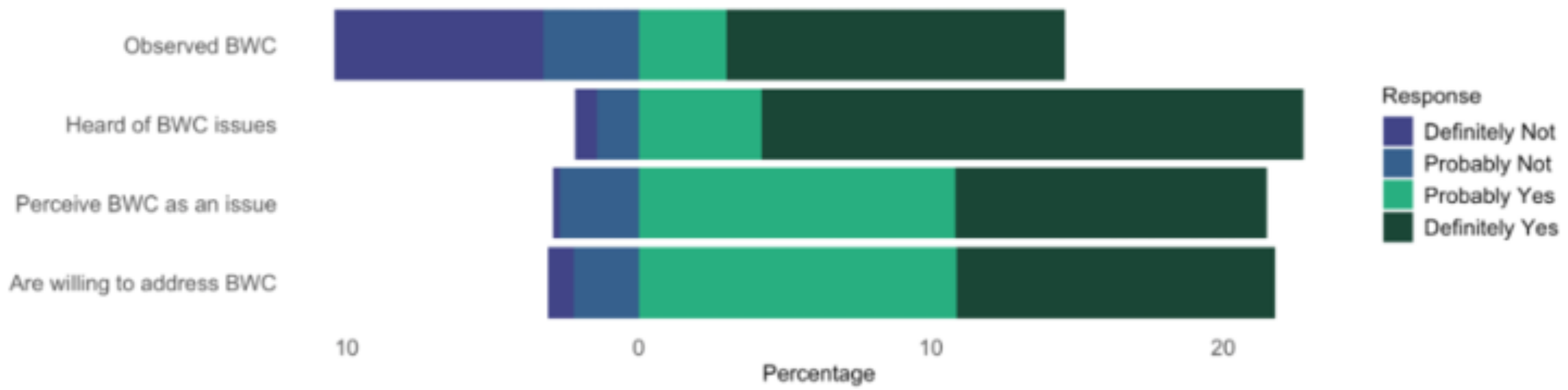

Figure 2

Click here to access/download;Figure;Fig2.tif

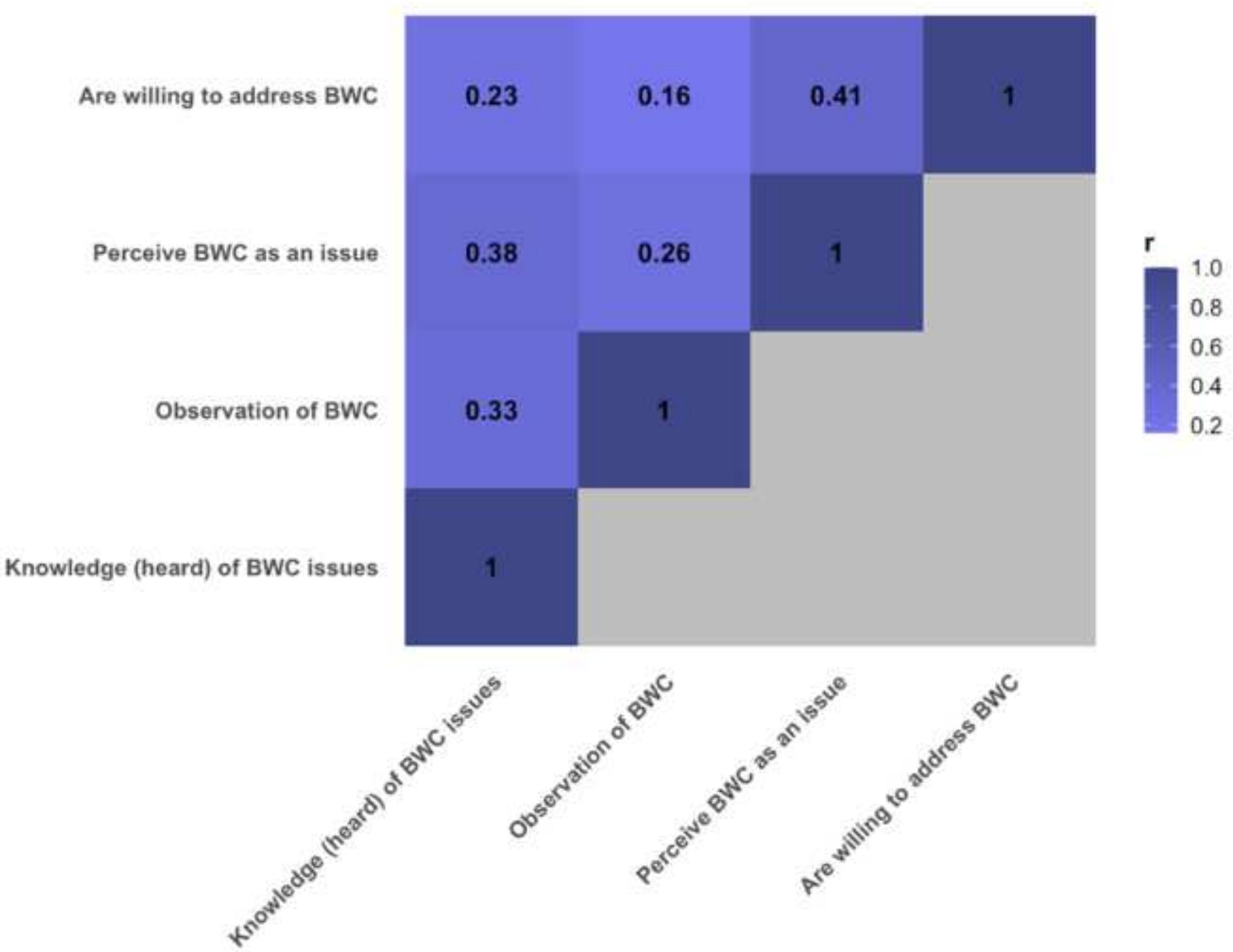

Figure 3

Click here to access/download;Figure;Fig3.tif

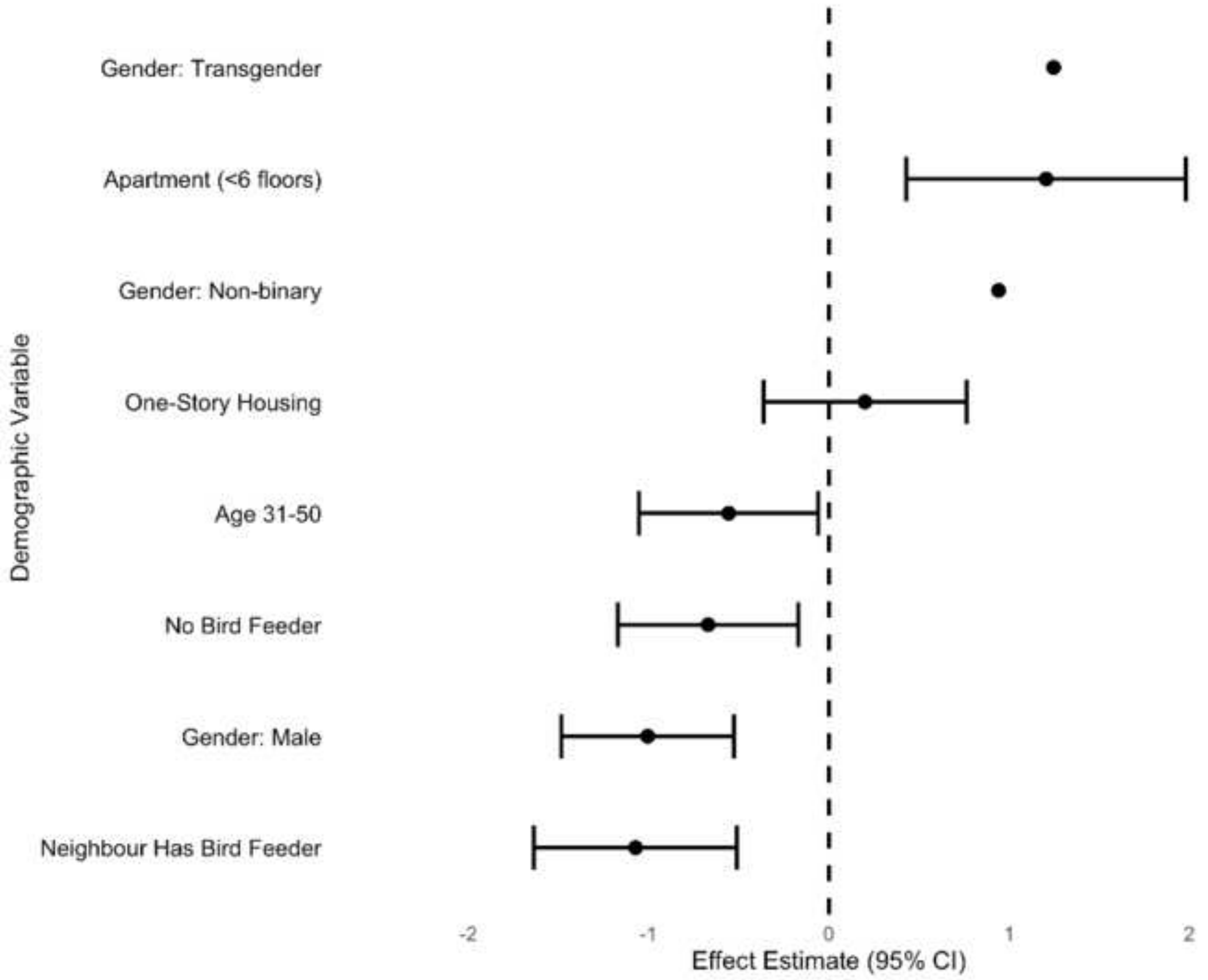

Figure 4

Click here to access/download;Figure;Fig4.tif

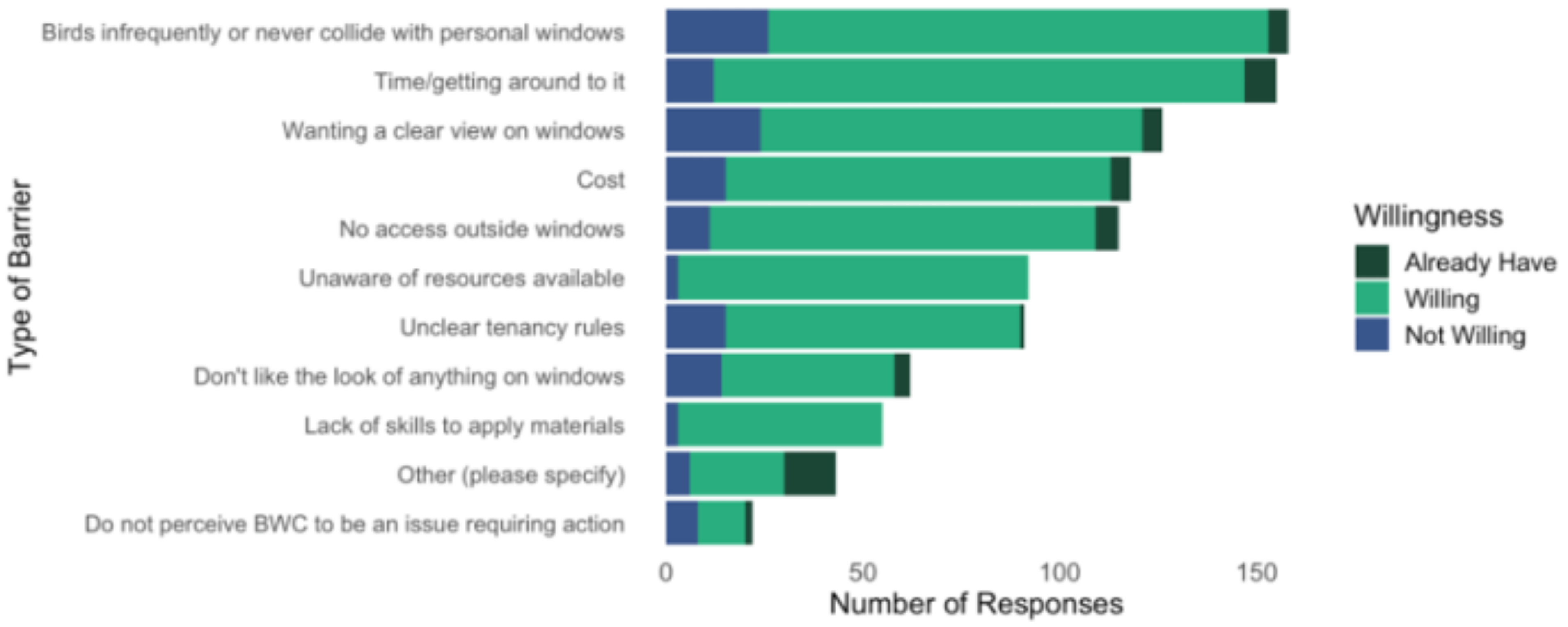

Figure 5

Click here to access/download;Figure;Fig5.tif

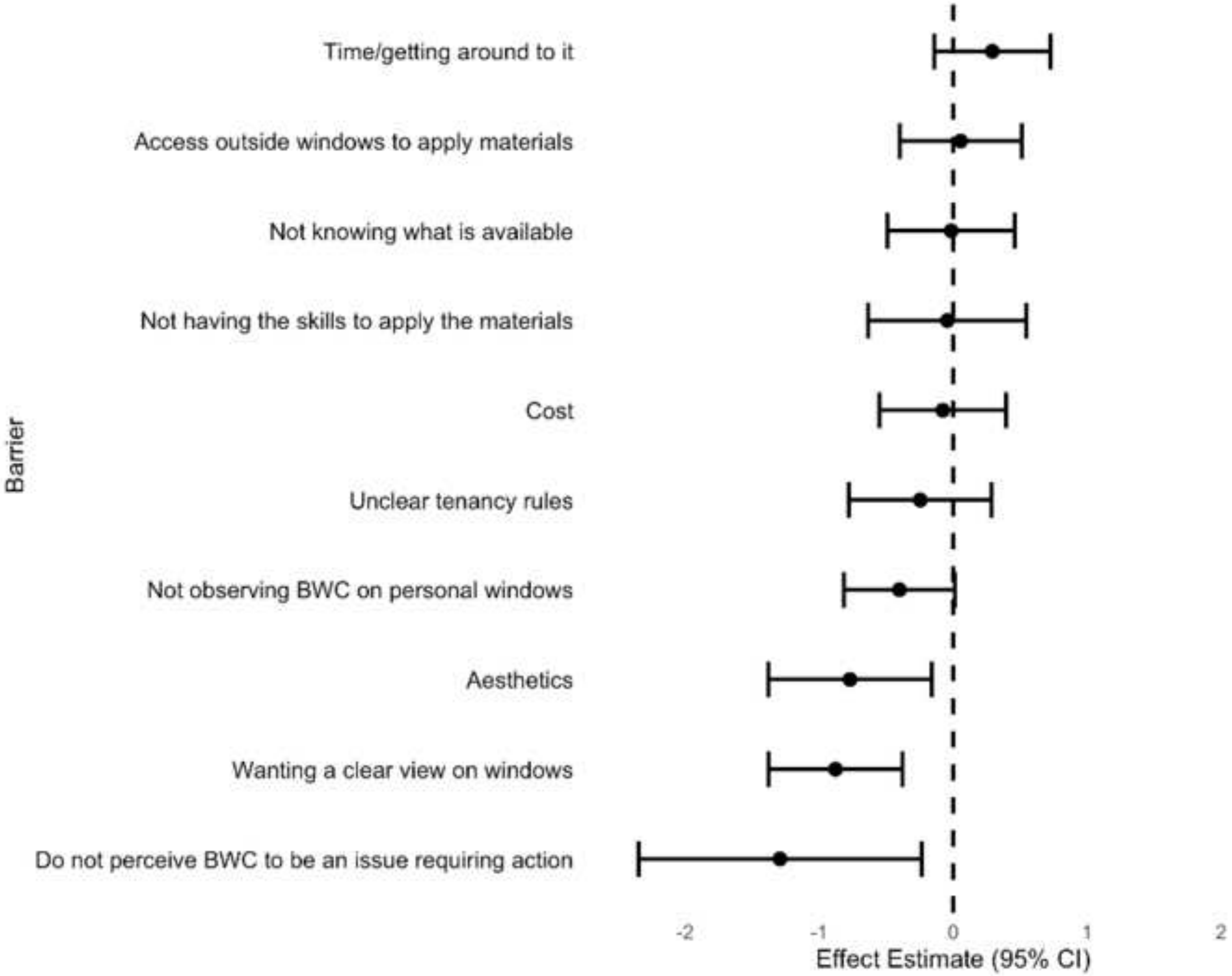

Figure 6

Click here to access/download;Figure;Fig6.tif

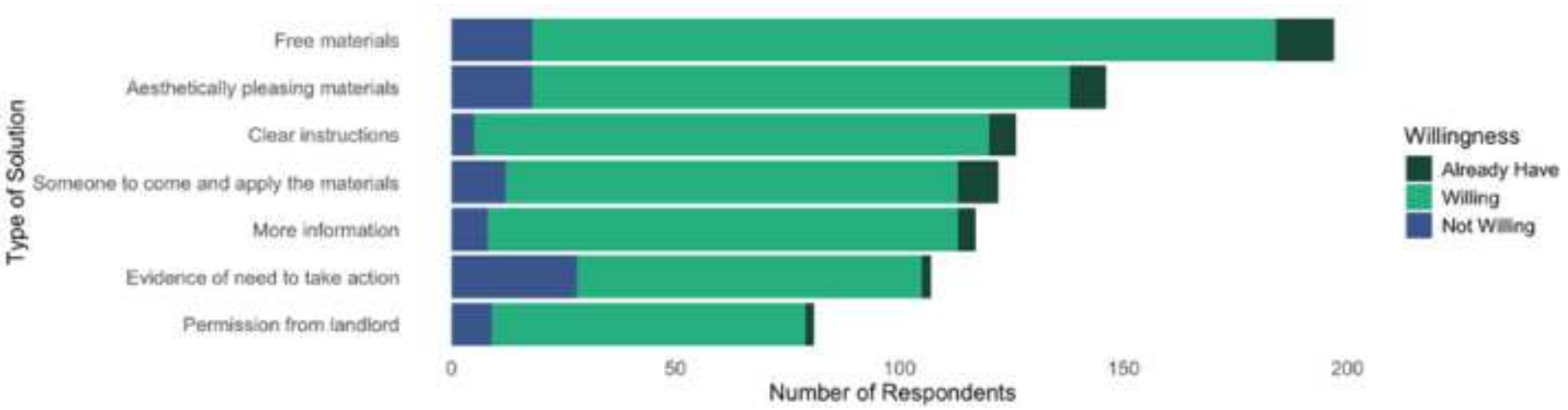

Figure 7

Click here to access/download;Figure;Fig7.tif

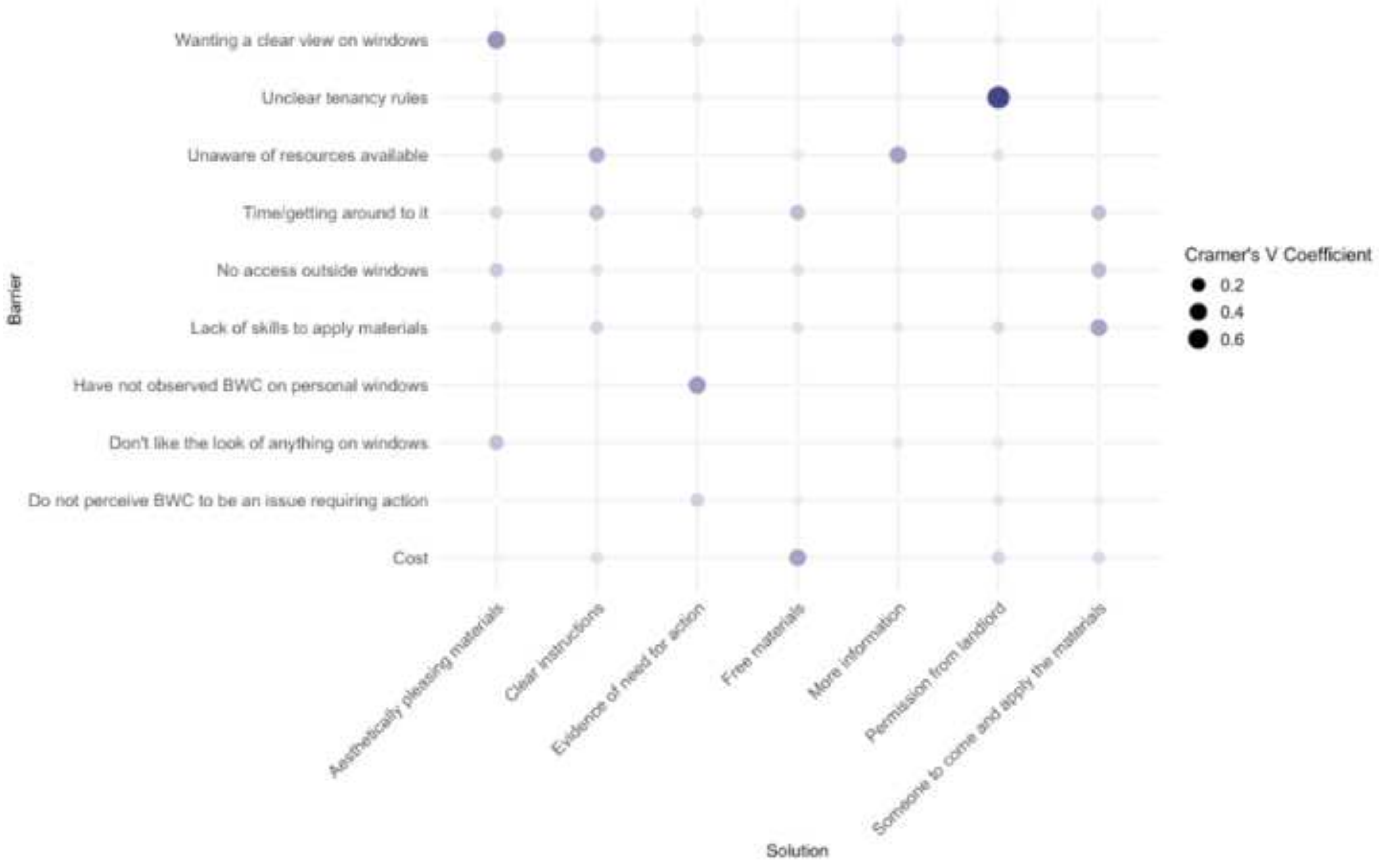

Click here to access/download
Supporting Information
S1 Survey.docx

Click here to access/download
Supporting Information
S2 Table.docx

Click here to access/download
Supporting Information
S3 Table.docx
