# Supplementary material for: Barriers and opportunities to preventing residential bird–window collisions
Source: PLoS One. 2026 Feb 25;21(2):e0342330. doi: 10.1371/journal.pone.0342330 (PMC12935251; doi:10.1371/journal.pone.0342330)
Supplement: Attachment — Submitted filename: Response to Reviewers_BWC_nov26.docx [file pone.0342330.s005.docx]

**Author Response to Editor**

Dear Dr. Lazebnik,

Thank you for the helpful feedback on our manuscript. Please extend our thanks to the reviewers as well, if possible.

Below, we have detailed how we revised the manuscript to address each reviewer’s comments, suggestions, or concerns. Our response to each point is typed in **bold** and references to the text are included in *italics* . Line numbers match those in the clean version of the revised manuscript with each change marked in the track-changes version. In regards to the funding statement and acknowledgements, we do not wish to change the funding statement but have revised the acknowledgements in the manuscript.

We hope you will find the revised manuscript acceptable for publication, and we look forward to hearing from you.

Sincerely,

Anastasia Lysyk & Aalia Khan, and all Co-Authors.

Academic editor comments:

When submitting your revision, we need you to address these additional requirements.

1. Please ensure that your manuscript meets PLOS ONE's style requirements, including those for file naming. The PLOS ONE style templates can be found at

<https://journals.plos.org/plosone/s/file?id=wjVg/PLOSOne_formatting_sample_main_body.pdf> and [https://journals.plos.org/plosone/s/file?id=ba62/PLOSOne_formatting_sample_title_authors_affiliations.pdf](https://can01.safelinks.protection.outlook.com/?url=https%3A%2F%2Ftrack.editorialmanager.com%2FCL0%2Fhttps%3A%252F%252Fjournals.plos.org%252Fplosone%252Fs%252Ffile%253Fid%3Dba62%252FPLOSOne_formatting_sample_title_authors_affiliations.pdf%2F1%2F010f0198f71fd847-9dfc96a6-3509-44fb-ab19-2d3a61e9a75c-000000%2Fmv27bmQiziDTg6iifEjXOoNMzF5KMcFePAemphi1s6o%3D226&data=05%7C02%7CStashaLysyk%40cmail.carleton.ca%7C12d38b25409d47a6ddc508ddec7e5be6%7C6ad91895de06485ebc51fce126cc8530%7C0%7C0%7C638926749669148537%7CUnknown%7CTWFpbGZsb3d8eyJFbXB0eU1hcGkiOnRydWUsIlYiOiIwLjAuMDAwMCIsIlAiOiJXaW4zMiIsIkFOIjoiTWFpbCIsIldUIjoyfQ%3D%3D%7C0%7C%7C%7C&sdata=B1OXRkZTcqLULRuNYq7c5w%2FUAFSaeBJK88TT1%2BF5wr4%3D&reserved=0)

**Response: We have made edits to meet the style requirements including maintaining sentence case in section headings, and bolding the figure titles in each figure caption.**

2. Thank you for stating the following in the Acknowledgments Section of your manuscript:

[We would like to thank A. Keller-Herzog, P. MacDonald, and others at the Community Associations for Environmental Sustainability, J. Niwa and W. English at Safe Wings Ottawa, and D. Doherty at Bird-friendly Ottawa for supporting this project. We thank community association representatives W. Burpee and P. MacDonald (FHACA), J. Freeman and T. Beauchamp (CGA), R. Philar (HCA), and D. Chapman (WCA), and the SOCI/ANTH 2180 class for recruiting survey respondents. RTB, AJVL, and AK were funded by NSERC (RGPIN 04888), the Kenneth Molson Foundation, and Environment and Climate Change Canada (GCXE24S042).]

We note that you have provided funding information that is currently declared in your Funding Statement. However, funding information should not appear in the Acknowledgments section or other areas of your manuscript. We will only publish funding information present in the Funding Statement section of the online submission form.

Please remove any funding-related text from the manuscript and let us know how you would like to update your Funding Statement. Currently, your Funding Statement reads as follows:

[Funding was provided to RTB, AJVL, and AK through three funding sources.

1) Natural Science and Engineering Research Council of Canada grant (RGPIN 04888) (https://www.nserc-crsng.gc.ca/index_eng.asp).

2) the Kenneth Molson Foundation (https://fondationmolson.org/en/).

3) Environment and Climate Change Canada (GCXE24S042) (https://www.canada.ca/en/environment-climate-change.html).

Funders did not play any role in study design, data collection and analysis, decision to publish, or preparation of the manuscript.]

Please include your amended statements within your cover letter; we will change the online submission form on your behalf.

**Response: We have removed the funding statement from the acknowledgements section of the revised manuscript.**

3. We note that your Data Availability Statement is currently as follows: [All relevant data are within the manuscript and its Supporting Information files.]

Please confirm at this time whether or not your submission contains all raw data required to replicate the results of your study. Authors must share the “minimal data set” for their submission. PLOS defines the minimal data set to consist of the data required to replicate all study findings reported in the article, as well as related metadata and methods (https://journals.plos.org/plosone/s/data-availability#loc-minimal-data-set-definition).

For example, authors should submit the following data:

- The values behind the means, standard deviations and other measures reported;

- The values used to build graphs;

- The points extracted from images for analysis.

Authors do not need to submit their entire data set if only a portion of the data was used in the reported study.

If your submission does not contain these data, please either upload them as Supporting Information files or deposit them to a stable, public repository and provide us with the relevant URLs, DOIs, or accession numbers. For a list of recommended repositories, please see <https://journals.plos.org/plosone/s/recommended-repositories>.

If there are ethical or legal restrictions on sharing a de-identified data set, please explain them in detail (e.g., data contain potentially sensitive information, data are owned by a third-party organization, etc.) and who has imposed them (e.g., an ethics committee). Please also provide contact information for a data access committee, ethics committee, or other institutional body to which data requests may be sent. If data are owned by a third party, please indicate how others may request data access.

**Response: We have updated the manuscript to include a DOI number to an online repository through Open Science Framework containing all relevant data and analyses (line 207).**

4. PLOS ONE now requires that authors provide the original uncropped and unadjusted images underlying all blot or gel results reported in a submission’s figures or Supporting Information files. This policy and the journal’s other requirements for blot/gel reporting and figure preparation are described in detail at <https://journals.plos.org/plosone/s/figures#loc-blot-and-gel-reporting-requirements> and <https://journals.plos.org/plosone/s/figures#loc-preparing-figures-from-image-files>. When you submit your revised manuscript, please ensure that your figures adhere fully to these guidelines and provide the original underlying images for all blot or gel data reported in your submission. See the following link for instructions on providing the original image data: <https://journals.plos.org/plosone/s/figures#loc-original-images-for-blots-and-gels>.

In your cover letter, please note whether your blot/gel image data are in Supporting Information or posted at a public data repository, provide the repository URL if relevant, and provide specific details as to which raw blot/gel images, if any, are not available. Email us at plosone@plos.org if you have any questions.

5. Please note that PLOS One has specific guidelines on code sharing for submissions in which author-generated code underpins the findings in the manuscript. In these cases, we expect all author-generated code to be made available without restrictions upon publication of the work. Please review our guidelines at <https://journals.plos.org/plosone/s/materials-and-software-sharing#loc-sharing-code> and ensure that your code is shared in a way that follows best practice and facilitates reproducibility and reuse.

**Response: Access to code sharing is included in our online repository through OSF.**

6. Please provide additional details regarding participant consent. In the ethics statement in the Methods and online submission information, please ensure that you have specified (1) whether consent was informed and (2) what type you obtained (for instance, written or verbal, and if verbal, how it was documented and witnessed). If your study included minors, state whether you obtained consent from parents or guardians. If the need for consent was waived by the ethics committee, please include this information.

If you are reporting a retrospective study of medical records or archived samples, please ensure that you have discussed whether all data were fully anonymized before you accessed them and/or whether the IRB or ethics committee waived the requirement for informed consent. If patients provided informed written consent to have data from their medical records used in research, please include this information.

**Response: We have described in the methods section that ethics were approved to describe informed participant consent through a survey question and all participants over the age of 16 were allowed to consent their own participation in the survey (lines 135-139).**

7. You indicated that you had ethical approval for your study. In your Methods section, please ensure you have also stated whether you obtained consent from parents or guardians of the minors included in the study or whether the research ethics committee or IRB specifically waived the need for their consent.

If the reviewer comments include a recommendation to cite specific previously published works, please review and evaluate these publications to determine whether they are relevant and should be cited. There is no requirement to cite these works unless the editor has indicated otherwise.

**Response: The ethical approval for this study allowed all participants over the age of 16 to confirm their own informed consent to participate in the survey (lines 135-139).**

Reviewer #1: This article summarizes a survey conducted in Ottawa, Canada, about bird-window collisions, which are a major cause of bird deaths in North America. The survey found that most Ottawa residents are aware of and concerned about bird-window collisions, and a high percentage are willing to treat their windows to prevent them. However, several factors act as barriers to implementing window treatments, especially the perception of infrequent collision and aesthetic concerns about how window treatments look and affect clear views as well as lack of time and costs associated with treating windows. The study suggests that offering free materials, aesthetically pleasing options, and clear instructions could help overcome these barriers. Ultimately, the authors advocate for targeted public messaging to highlight the impact of residential homes on this issue and to encourage widespread adoption of bird-friendly window treatments.
The article is interesting, well written and succinct. I provide comments below to help clarify the writing and the presentation of results and subsequent discussion.

**Response: Thank you very much for this positive feedback!**

Major comments:
- Are the legends correct on Figures 4 and 6? I ask because I don’t understand the “already have” for many of the responses such as “no access” (+ others) and what that has to do with willingness. Also, in the methods, I thought you asked the questions on a 5-pt Likert Scale but the answers aren’t presented that way. Could you please clarify all this where needed.

**Response: We have clarified these are three separate responses by removing “***willingness***” in the legend title and we have changed “***already have***” to “***already have taken action to address BWC***” in Fig.4. In the same figure, we have changed “***no access outside windows***” to “***no access to outside windows***”.**

- Paragraph that starts at L340. There is quite a bit of literature about the gap between environmental knowledge and pro-environmental action. I suggest integrating some of that literature into this paragraph or a new one so that you can tie your results to theoretical models of this “gap”. This is one key paper along those lines: <https://www.tandfonline.com/doi/abs/10.1080/13504620220145401> but there are others.

**Response: Thank you for suggesting this paper, we have incorporated this and others in the revised paragraph starting on line 362.**

- Are the mixed models fitted with all the respondents or just the ones who do not already have treatments on their windows? If the former, when you run the models with the subset, do the results change? What about for the correlations?

**Response: Thank you for this suggestion. We agree with your feedback and have decided to remove respondents who already have treatments on their windows from our models and correlation analysis, as they have already bypassed the barriers we are exploring in our study.**

**Results do not change substantially when the models are fitted with the subset, with the exception that people aged 31-50 are no longer significantly less willing to act compared to reference (age 50+). Fig2, Fig3, Fig5, Table S2 and Table S3 have been updated with the new results.**

- In the methods, you say that socio-economic and demographic questions were optional on the survey, what is the effective response rate for those questions, and how many respondents are included in the statistical models you build later? I believe this should be included at the very least in the figure captions, maybe as n=XXX).

**Response: Thank you for your suggestion. We have included the response rate and number of respondents used for our statistical models in our methods section (line 195-200).**

- L183, will need to be changed to the link to the OSF repository upon publication

**Response: The DOI number to the OSF repository has been added to the manuscript (line 209).**

- The references are not formatted properly (e.g. journal titles should be italicized) and some references are missing information (e.g. 12 and 21). Please check those thoroughly or use a formatting software such as Zotero (free).

**Response: Thank you for your feedback. We have edited the references according to existing guidelines for PLOS ONE (journal titles are not italicized as the citation style follows the “Vancouver” format). We have added the missing information.**

Minor comments:
- For future surveys you might conduct, Dillman et al. (<https://sesrc.wsu.edu/about/total-design-method/>) recommends writing questions as statements and then using the standard definitely agree to disagree Likert scale. So your questions about “Are you willing to not willing to X”, become “I am willing to X”.

**Response: Thank you for the suggestion, which we will consider in future studies.**

- For Figure 2, in the grey squares, I would put the p-values or have some way to visualize them. This might work for you but is not the only way to do this: <https://indrajeetpatil.github.io/ggstatsplot/articles/web_only/ggcorrmat.html> You could also just add the p-value in parenthesis in the colored box for ease of interpretation (or present this Figure as a table which may be more efficient)

**Response: Thank you for this suggestion! We have now included p-values in the colored boxes for Figure 2.**

- Line 67, could you specify what a low-rise building is?

**Response: Addressed in line 66.**

- Line 84, missing “on”

**Response: Addressed.**

- Line 97 at the end, missing “and”

**Response: Addressed.**

- Line 110, in my field, the word data is treated as a plural (datum is the singular) so it would be “data were” but I’ve noticed this seems to be journal/field specific.

**Response: Addressed.**

- Line 123, spell out “4” as “four”. I think anything below 12 is expected to be spelled out (occurs on L 157 and 160, 192 too).

**Response: Changed here and throughout the paper.**

- L163, it would be helpful to have the references for the literature that informed the list of barriers and solutions (assuming it’s <~6 refs)

**Response: Thank you, we have included references at the end of this sentence (Line 180).**

- L172, instead of “ran” consider “fit” and the order of the sentence could be different: you fit the model Mixed Effect Model with a cumulating link and Laplace approx..

**Response: Changed**.

- L212, what is the p-value for the observation and knowledge questions?

**Response: Added to results.**

- L219-220, I don’t see that info in Figure 1.

**Response: Addressed - Fig1 is no longer cited for this info.**

- L219 and 225, you repeat the same info about already treated households.

**Response:** **Removed repeated line.**

- L227-231 seem to be a repeat of L206-212 as well. Pick one or merge the two perhaps?

**Response: Thank you for your suggestion. Results on L206-212 (now 233-238) are about correlations among knowledge, observation, and perception, while results on L227-231 (now 254-258) are about correlations with willingness. We have removed the term *“****willingness”* **(line 231-232) to make this more clear.**

- L237, what does PE stand for?

**Response: PE stands for “parameter estimates” - we have defined this term in the methods section in lines 200-201.**

- L235, consider replacing “also affected” by “were also associated with” since the first implies causation

**Response: Addressed**.

- L320, consider replacing “older” with “middle-aged” or “over 50 years old”?

**Response: Addressed**.

- L320, I think you need to rephrase that sentence of maybe replace the “and” with a “who” or “who identified with”

**Response: Addressed**.

- Sentence starting at L322 has too many “ands”.

**Response: Changed to remove an “and”**

- L325, results don’t find anything, please rephrase

**Response: Changed to “we found”**

- L327-328, you say multiple studies corroborate your findings, but only cite one (32). Either chance studies to study or cite more than one study.

**Response: Additional citations added in line 368.**

- L332, at “Respondents”, consider making that a new paragraph.

**Response: This section of the discussion has been thoroughly revised and the new edits should reflect this writing comment.**

- In a future study, it might be interesting to about observed collisions where the bird(s) died upon impact or not.

**Response: Thank you for the suggestion! Samuels et al. (2022) conducted a similar study to this suggestion which we have cited in our discussion (lines 403-407) regarding how many collisions are not observed at homes.**

Reviewer #2: This manuscript describes survey-based research study in which a sample of citizens from various neighborhoods in Ottawa, Canada were surveyed about their knowledge and perception of bird–window collisions at residential homes, their willingness to treat windows to prevent collisions, and barriers to action. The survey resulted in several significant correlations between variables, including perception of bird–window collisions and willingness to treat windows. Somewhat surprisingly, perception of collisions as a problem that should be addressed was more significantly correlated with willingness to act than experience witnessing a bird–window collision. Another important finding is that many respondents had witnessed a collision at home but needed more evidence that such collisions are a big enough problem before they would consider treating their windows. Significant barriers to respondents treating their windows at home included time, cost, and lack of clear instructions. All these points can help inform conservation groups and collision monitoring programs tailor their educational, outreach, and advocacy efforts to better communicate that most collisions occur at low-rise residential buildings rather than at tall downtown buildings that are often featured in news media as being the top cause of collisions.

Considering the great numbers of birds that die each year from building collisions in North America alone, this study has much merit, as it helps fill a gap in the published literature and should aid conservationists and communications experts working to spread awareness of and reduce bird–building collisions. As an ecologist and researcher with years of experience leading a collision monitoring program, I was excited to see this study and look forward to further studies that will build off this.

The manuscript is well-written overall, presented in logical order, and includes sound statistical analyses, useful supplementary tables, and relevant references. I recommend accepting the manuscript, pending revisions.

**Response: Thank you for the positive feedback!**

Major Feedback:

1) While the authors mention some limitations of their survey in the Discussion, the Methods section does not describe any steps that were taken to reduce bias in data collection. This is a serious concern, as survey results are highly sensitive to issues such as sampling bias, non-response bias, and social desirability bias. I recommend that the authors provide more detail on how the survey was designed and administered to minimize these risks (e.g., question design, question randomization, recruitment strategy, etc.). Without this information, it is difficult to evaluate the reliability of the findings.

**Response: Thank you for raising this important point. We have added to the methods section describing how the survey was created and circulated through the undergraduate course. We have also described that the questions were designed to reduce social desirability bias and avoid the perception of a preferred response (lines 40-48).**

2) For readers who are not familiar with Likert scale charts (like me), Figure 1 may be difficult to understand as presented. I recommend adding more detailed explanations of how to interpret this to avoid confusion and perhaps add percentages inside the bars. For example: explain why Fig. 1 has positive and negative percentages, why the bars are staggered, and what this means.
**Response: Thank you for your suggestions. We have added percentages on top of bars, added a line at 0 to make the staggering appear more intuitive, and edited the figure caption (lines 226-232) to include** *“Negative values (i.e, values to the left of zero) represent the percentage of respondents who selected “definitely not” or “probably not”. Positive values represent “probably yes”, “definitely yes” or “already have”***, to reflect this feedback.**

**We made an additional change while editing this figure. Originally, each percentage was calculated by dividing by the *total* number of respondents; this has been changed so that now each is calculated by dividing by the number of respondents in each category.**

3) The use of CLMMs is appropriate for ordinal responses, and I appreciate that the authors ran two separate models and presented the results in forest plots. However, the basis for Figure 3 is not entirely clear. From Table S2, it appears that the demographic model used ‘>50 years’, ‘2–4 story house’, ‘female’, and ‘owns a bird feeder’ as reference categories, but this is not explained in the text. The reference categories should be stated clearly in the main text or Fig. 3 caption so readers can interpret the effect estimates in relation to the correct baseline groups.

**Response: Thank you for your comment. Reference categories have been added to figure captions (lines 279-286). Results have also been grouped by demographic category for further visual clarity.**

In addition, one statistically significant covariate (age 16-30) is not included in Fig. 3, whereas two non-significant covariates (transgender, non-binary) are included. In contrast, Fig. 5 displays all predictors, regardless of significance. I recommend clarifying the rationale for which covariates are displayed in each forest plot and ensuring consistency in presentation. Otherwise, readers may incorrectly assume that omitted variables were not significant or were not tested.

**Response: Thank you for your feedback. A code bug resulted in the age 16-30 covariate to be missing from the figure - it has been added to Fig. 3. The transgender and non-binary categories have been removed from analysis altogether as there were too few data points.**

Lastly, I recommend including an explanation of what the parameter estimates mean in the main text for clarity. Currently, this information is only stated in Tables S2 & S3.

**Response: We have added a description of parameter estimates in the main text: *“****Forest plots were created to depict the associations of each variable on the response as parameter estimates (PE) with 95% confidence intervals.*” **(lines 200-201). We have also included this in the captions for figures 3 and 5.**

4) The Discussion section is quite wordy and restates much information presented in the Results section, making it unnecessarily long and repetitive. Revising for conciseness, focusing on what the results mean and why this should matter to readers, without restating the results, would make the discussion much stronger and more digestible/useful for readers.

**Response: Thank you for this suggestion. We have thoroughly revised our discussion to avoid repetition and streamline our writing.**

Minor Feedback:

Minor suggestions regarding grammatical errors, typos, etc. can be found as comments in the attached pdf. Other minor feedback is listed below.

1) Throughout the manuscript, the authors use a hyphen (-) between “bird” and “window” when referring to collisions, but an en dash (–) should be used instead. Hyphens should be used in compound adjectives, e.g. bird-friendly materials, whereas an en dash should be used to indicate linkages between two things, e.g. bird–window collisions.

**Response: Addressed throughout the paper.**

2) Ln 38-40: It isn’t helpful to report parameter estimates in the abstract, as readers cannot interpret these outside the context of the full model results. I recommend highlighting the meaning of the results (relative strength, significance) rather than raw statistical values in the abstract.

**Response: Thank you for this feedback. We have removed parameter estimates from the abstract.**

3) Ln 65-66: The article cited here (Klem & Saenger 2013) isn’t about window films or decals. They tested UV-treated windows and Acopian BirdSavers in this study. There are several better options to cite here, including DeGroot et al. 2022 (doi: 10.7717/peerj.13142) or Bird-friendly Building Design from American Bird Conservancy (https://abcbirds.org/wp-content/uploads/2015/05/Bird-friendly-Building-Guide_2015.pdf).

**Response: Thank you, we have added citations to this line (lines 63-64).**

4) Ln 182-183: Please add an accession number or URL prior to publication.

**Response: The link to the OSF repository has been added to the manuscript in line 209.**

5) Ln 186-195: It appears that the percentages reported in this paragraph are based on all 465 respondents rather than the 422 included in the analysis. For clarity and consistency throughout the Results section, please base these percentages on the number of responses used in the analysis.

**Response: We have updated percentages to reflect the number of responses used in analyses.**

6) Ln 187-189: It is unclear if the percentages of older respondents and those who identified as white are based on the number of female respondents only or all respondents. Please reword to clarify.

**Response: Thank you for this feedback. We have streamlined and reworded our results to include (X% *of all respondents*) for clarification.**

7) Ln 189-190: It is unclear why counts are reported here rather than percentages. I recommend reporting percentages for consistency. Again, these should be based on the number of responses used in the analysis.

**Response: We have removed counts from these lines and used percentages of responses used in the analysis.**

8) Ln 212: For consistency, please report the p-value with the correlation coefficient, as in line 209.

**Response: Thank you for this feedback. We have changed our results to report p-values with all correlation coefficients.**

9) Ln 219-220, 224-225: The percentage of respondents who had already treated their windows is reported twice in this paragraph. I recommend deleting the last sentence and removing the word “only” from the first sentence.

**Response: Addressed, as also suggested by reviewer #1.**

10) Figure 3: To aid in comparing the importance of variables within categories, I recommend reordering the variables to be grouped by category rather than strictly in increasing numeric order. I also recommend denoting which variables are significant to aid readers who may be less familiar with interpreting forest plots.

**Response: We have explained significance in the figure captions with “***Estimates with confidence intervals that do not overlap with zero are significant”* **(lines 284-285). Variables are also now grouped by category.**

11) Ln 289: Please clarify what is meant by “more information”. (More information about what?)

**Response: We have changed this to “***more information on treatment options***” on line 328**

12) Figure 5: As with Fig. 3, I recommend denoting which barriers are significant.

**Response: Thank you for this feedback - we have added “***Estimates with confidence intervals that do not overlap with zero are significant”* **to the caption for Figure 5 (lines 321-322) as well.**

13) Ln 320-328: Considering that the authors reported in the Results that the largest proportion of respondents were older, white females, I wonder if this may introduce bias into the survey results and interpretation via sampling bias. Thus, I would like to know if the authors took this into consideration.

**Response: Thank you for raising this point. We did not consider this to introduce sampling bias and we discuss how this demographic of respondents more frequently responds to surveys and are generally more receptive to acting on environmental issues as described in other studies (lines 365-369).**

14) Ln 333-336: It is not clear if the authors are referring to all respondents or a subset of respondents here, nor why this group is considered more likely to act. Please clarify. Also, the end of this sentence reads clumsily. I suggest revising to something like, “…suggesting barriers that prevent people from taking action still exist.”

**Response: We have thoroughly revised this section of the discussion in the paragraph starting on line 370.**

15) Ln 362-363: I suggest changing “under” to “nearly” for clarity.

**Response: Changed.**

16) Ln 399: I suggest inserting “frequently” before “followed”, as carcasses are not always depredated.

**Response: Addressed.**

17) Ln 426-427: This is true, but considering the survey results, it seems this will only lead to a significant number of individuals treating their windows if the monetary aid is in conjunction with suggestions for bird-friendly products with clear, simple instructions (such as FeatherFriendly). I feel this is worth mentioning.

**Response: Added this suggestion to lines 446-451.**

18) Ln 447-450: The way this sentence reads, it suggests that such a shift has already occurred, rather than a shift is needed. I suggest revising as, “…they also suggest a shift is needed in the type…”.

**Response: Addressed in line 472**

19) Ln 457: Perhaps replace “a few” with “some”, as this makes it seem that very few collisions occur at individual homes.

**Response: This line has been rephrased.**

20) Ln 461-463: An excellent resource that could be included as an example here is the Glass Collisions section of American Bird Conservancy’s website, particularly their Products & Solutions Database (<https://abcbirds.org/glass-collisions/products-database/>).

**Response: Information added and included the FLAP Canada resource page as a Canadian source in lines 486-487.**

21) References: It would benefit readers to include DOI numbers for referenced articles.

**Response: Thank you for this suggestion. We have included DOI numbers for all referenced articles for with a DOI is available.**

22) Ln 510-511: Please correct the formatting for the Warren (2013) reference, as it is a Master’s Thesis.

**Response: Addressed.**
